# Activated neutrophil fluorescent imaging technique for human lungs

**DOI:** 10.1038/s41598-020-80083-w

**Published:** 2021-01-13

**Authors:** Thomas H. Craven, Tashfeen Walton, Ahsan R. Akram, Emma Scholefield, Neil McDonald, Adam D.L. Marshall, Duncan C. Humphries, Bethany Mills, Thane A. Campbell, Annya Bruce, Joanne Mair, James W. Dear, David E. Newby, Adam T. Hill, Timothy S. Walsh, Chris Haslett, Kevin Dhaliwal

**Affiliations:** 1 Translational Healthcare Technologies Group, Queen’s Medical Research Institute, University of Edinburgh, 47 Little France Crescent, Edinburgh, EH16 4TJ UK; 2 Edinburgh Critical Care Research Group, University of Edinburgh, Edinburgh, UK; 3 School of Chemistry, EaStCHEM, University of Edinburgh, Edinburgh, UK; 4 Centre for Cardiovascular Science, University of Edinburgh, Edinburgh, UK

**Keywords:** Respiratory tract diseases, Cellular imaging

## Abstract

Neutrophil activation is an integral process to acute inflammation and is associated with adverse clinical sequelae. Identification of neutrophil activation in real time in the lungs of patients may permit biological stratification of patients in otherwise heterogenous cohorts typically defined by clinical criteria. No methods for identifying neutrophil activation in real time in the lungs of patients currently exist. We developed a bespoke molecular imaging probe targeting three characteristic signatures of neutrophil activation: pinocytosis, phagosomal alkalinisation, and human neutrophil elastase (HNE) activity. The probe functioned as designed in vitro and ex vivo*.* We evaluated optical endomicroscopy imaging of neutrophil activity using the probe in real-time at the bedside of healthy volunteers, patients with bronchiectasis, and critically unwell mechanically ventilated patients. We detected a range of imaging responses in vivo reflecting heterogeneity of condition and severity. We corroborated optical signal was due to probe function and neutrophil activation.

## Introduction

Neutrophils and neutrophil activation are a hallmark of inflammation^1–7^. Despite having a pivotal role in host defense, uncontrolled neutrophil activity leads to excessive tissue injury through proteinases, cytokines and reactive oxygen species^8,9^ and the induction of apoptosis in epithelial cells^10^. Phenotypic analysis of peripheral blood neutrophils, whilst useful, does not reflect the post-migratory phenotype of tissue neutrophils in vivo in situ*.* Currently, there are no sensitive and specific methods to detect in situ neutrophil activation in real-time.

Many diseases are characterised by excessive neutrophil activity. In the lung these would include acute respiratory distress syndrome (ARDS)^11,12^, community and ventilator associated pneumonia^3^, chronic obstructive pulmonary disease^13^, and bronchiectasis^14^. Detecting and monitoring real-time neutrophil activity in vivo in situ may offer useful insights into disease pathology. Furthermore, the ability to detect neutrophil activation at the bedside in mechanically-ventilated (MV) patients offers a potential clinical tool to aid stratification and therapeutic targeting in ARDS and its risk groups. In airway and parenchymal diseases characterised by neutrophilic inflammation and damage, we hypothesized that the combination of a specific optical SmartProbe and bedside optical endomicroscopy (OEM) would provide an in situ indicator of alveolar neutrophil activation.

Enzymatic activity is common target for investigation and lends itself to investigation through optical techniques. Neutrophil elastase is the most abundant enzyme released from activating neutrophils, and elastase specific probes have been used to examine the spatiotemporal function of elastase on the surface of cells^15,16^ and intracellularly^17^, and during in vivo murine modeling of lung injury^18^. To date, no imaging probe has been translated for in vivo human imaging, but with the development of fibre-based optical endomicroscopy there have been increasing deployments of optical imaging for lung applications at the bedside^19–22^. We previously reported a multi-branched compound for fluorescent detection of neutrophil elastase^23^ and have modified that structure to generate a bespoke chemical SmartProbe for the detection of activated neutrophils that would be suitable for clinical deployment.

This Neutrophil Activation Probe (NAP) consists of three internally quenched fluorescein moieties, each conjugated to an optimized peptide sequence, and all attached to a tri-branched multivalent scaffold. It was designed to amplify fluorescent emission and ‘tag’ neutrophils in response to three processes in the pathway of neutrophil activation; (i) enhanced pinocytosis, (ii) progressive alkalinization of the phagosome, and (iii) increased human neutrophil elastase (HNE) activity. Together, the targeting of these augmented metabolic processes makes the generated cellular fluorescent signal specific to activated neutrophils.

## Results

### NAP is a sensor of human neutrophil elastase activity and pH

**Figure 1:**
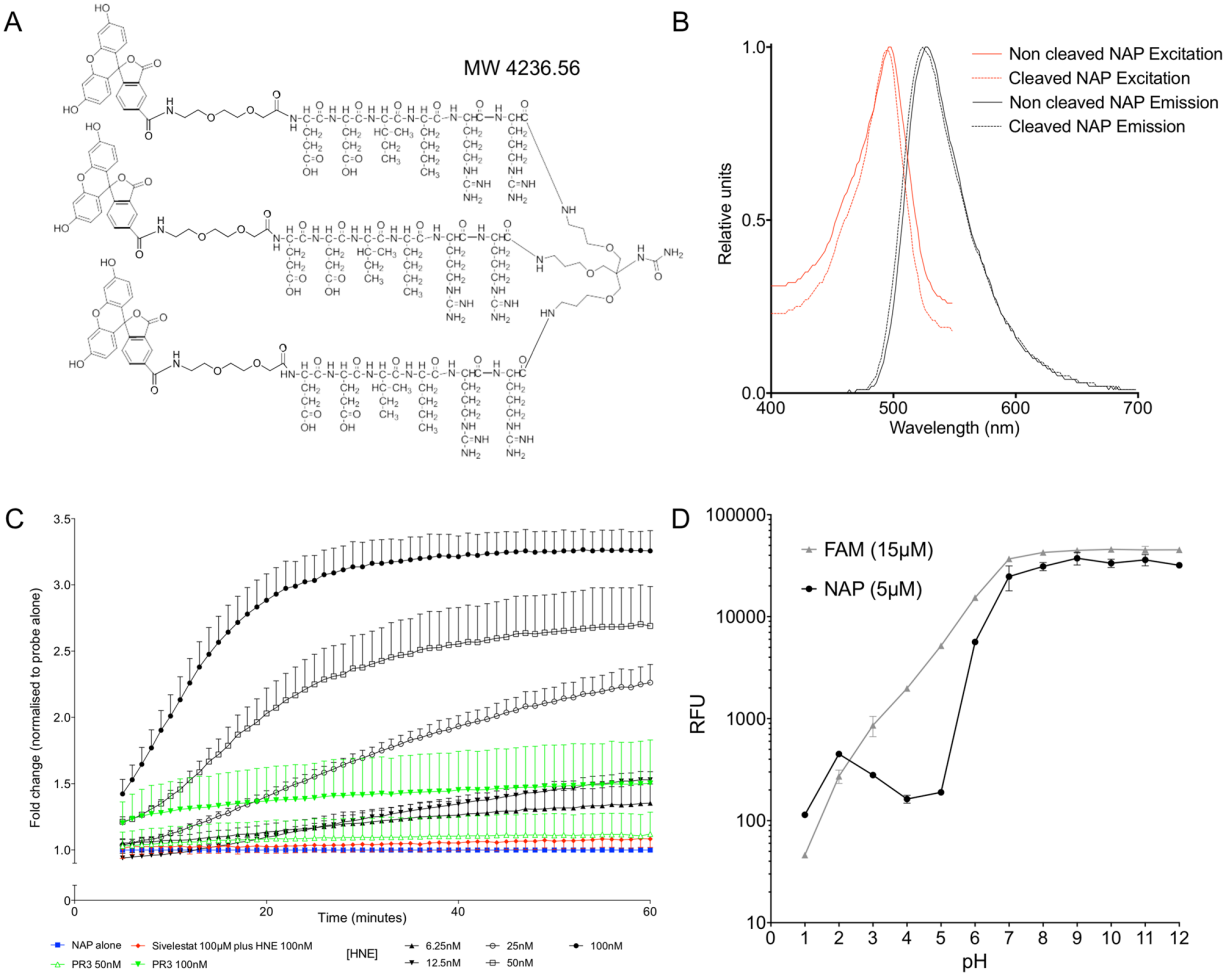
**(A)** Chemical structure of the NAP Smartprobe; **(B)** the absorbance spectra (with pH) and the emission spectra for NAP in phosphate buffered saline at pH 7.4 (5 µM, λ_ex_ = 430 nm). Cleaved in the presence of elastase, which liberates carboxyfluorescein from the dendrimer backbone, releases internal quenching mechanism and permits fluorescence. **(C)** Time course of fluorescence dequenching in the presence of purified HNE. Coloured lines represent control conditions (NAP in the absence of HNE, blue line; NAP and 100 nM HNE in the presence of the specific HNE inhibitor Sivelestat (100 µM), red line; NAP and 50 nM or 100 nM proteinase 3, green lines). Mean point estimate (+ SEM) from three independent experiments. Modest cleavage is shown with supraphysiological concentrations of proteinase 3, an alternative neutrophil specific serine protease. **(D)** Change in fluorescence with pH (freshly made up pH buffer). NAP (5 µM) shows an increase in fluorescence as pH increases (black circles). There is a steepening of the curve around physiologically relevant pH values. At equivalent concentrations of carboxyfluorescein (15 µM) there is the expected increase in fluorescence as H^+^ concentration falls (grey triangles). Mean point estimate (± SD, n = 1, in triplicate). (Note the concentration of NAP is 5 µM, but it carries 3 copies of carboxyfluorescein).

NAP consists of a six-amino acid (Glu–Glu–Ile–Nle–Arg–Arg) sequence on a tri-branched scaffold (Fig. 1A) with a λ_ex_ of 488 nm (Fig. 1B) ensuring compatibility with clinical OEM systems that employ the 488 nm argon ion-excitation source. When incubated in the presence of physiologically and pathologically relevant concentrations of HNE^3^, there was a rapid dequenching (Fig. 1C) with an increase in fluorescence of nearly 350% compared to the resting state. Cleavage was also completely abrogated in the presence of Sivelestat, a specific inhibitor of HNE^24^. NAP acted as a pH sensor, in keeping with a multitude of fluorescein protolytic forms, but with a serendipitous steepening of the fluorescent response as pH increases over the physiologically relevant range compared to the emission spectra of carboxyfluorescein (Fig. 1D), with the pH profile modulated by the tri-branched nature of the construct, thereby altering the pKa values expected for carboxyfluorescein.

### Enhanced cellular fluorescent signal is specific to activated neutrophils

**Figure 2:**
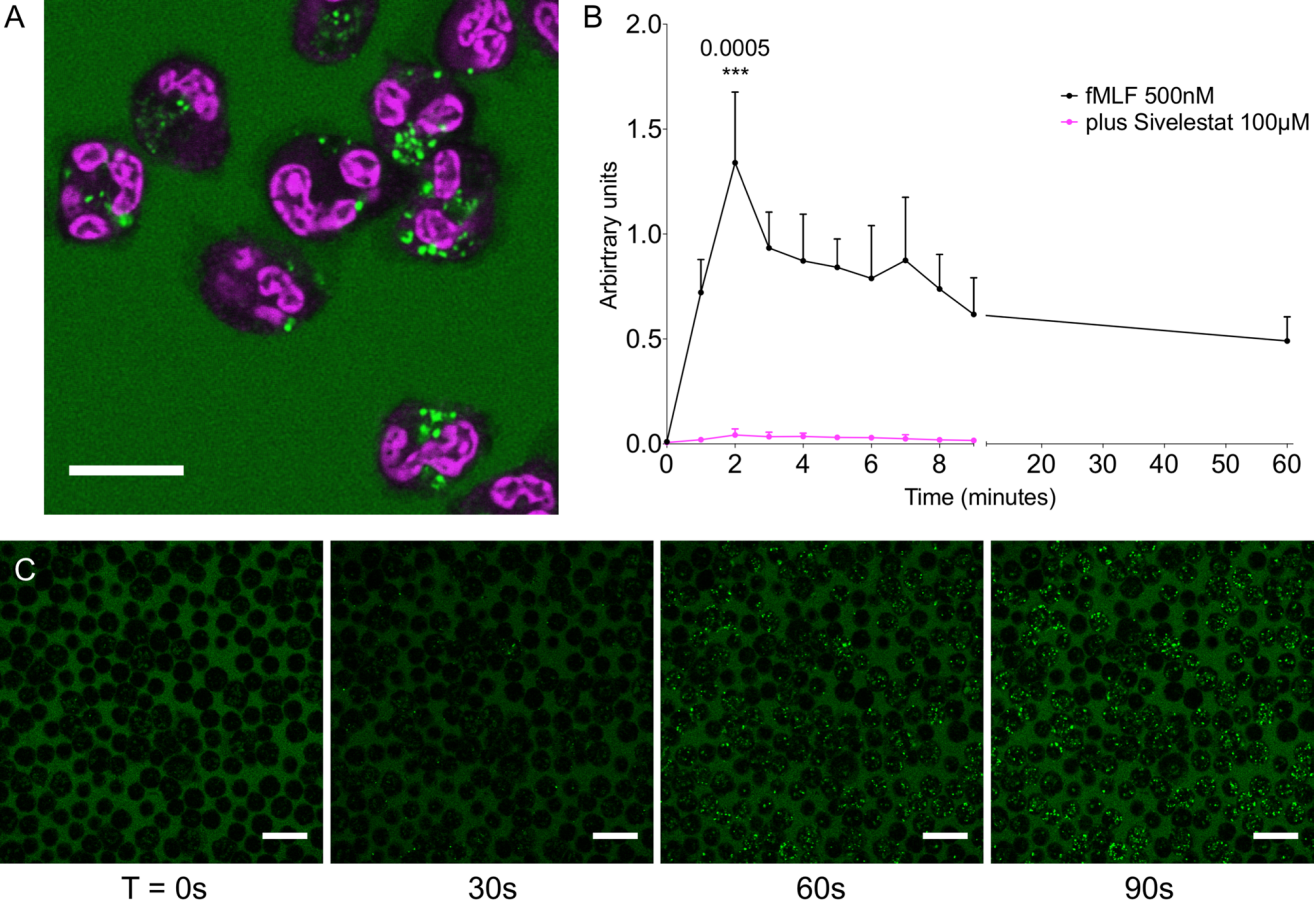
NAP selectively identifies activated neutrophils. **(A)** Activated neutrophils take up NAP (green) to exhibit bright green intracellular punctate fluorescent signal. Cells were stained with the nucleic acid counterstain Syto 60 (magenta). Scale bar 10 µm. **(B)** Graph illustrating results of objective image analysis pooled from three separate experiments (mean + SD). Dots are mean (± SD). Black line—primed neutrophils activated with fMLF (500 nM) at t = 10 s. Magenta line—primed neutrophils in continued presence of Sivelestat 100 µM, activated with fMLF (500 nM) at t = 10 s. Statistical analysis by 2-way ANOVA with Bonferroni’s correction, p = 0.0082 between conditions up to 9-min time point, 3 degrees of freedom, an exact multiplicity adjusted p value is given with the figure at t = 2 min for illustrative purposes. N = 3 (distinct samples) for fMLF condition, N = 2 (distinct samples) for Sivelestat condition. **(C) **Confocal microscopy images of human neutrophils activated using 500 nM fMLF shown at various time points in the presence of NAP (5 µM, green). Images are representative from three separate experiments. Scale bar 20 µm.

Confocal microscopy was used to characterise the imaging properties of NAP in vitro*.* Isolated neutrophils, treated with the calcium ionophore A23187 (10 µM, in order to increase intracellular calcium and induce a variety of secondary activation effects such as degranulation and superoxide production) in the presence of NAP (5 µM), showed an increase in intracellular punctate fluorescence compared to untreated neutrophils (Fig. 2A and Supplementary Fig. S1), while human alveolar macrophages retrieved from broncho-alveolar lavage fluid (BALF) and exposed to NAP ex vivo showed no signal when activated (Supplementary Fig. S2). Treated and untreated mixed leucocyte populations were analysed by flow cytometry, which confirmed that the NAP signal was specific to activated neutrophils compared to peripheral blood mononuclear cells and quiescent neutrophils (Supplementary Fig. S3).

### NAP dequenching is rapid and sustained

When isolated healthy human neutrophils, primed with Cytochalasin B (10.4 µM), were exposed to the chemotactic peptide N-Formyl-Met–Leu–Phe-OH (fMLF) (500 nM) in order to induce calcium mediated degranulation, the appearance of intracellular punctate fluorescence occurred rapidly, reaching a peak within two minutes and being sustained for at least one hour. This intracellular fluorescent signal was abrogated by co-incubation with Sivelestat (100 µM) (Fig. 2B,C and Supplementary video S1).

### NAP signal is dependent on dynamin-dependent internalization, HNE activity and alkalinisation of the neutrophil phagosome

**Figure 3:**
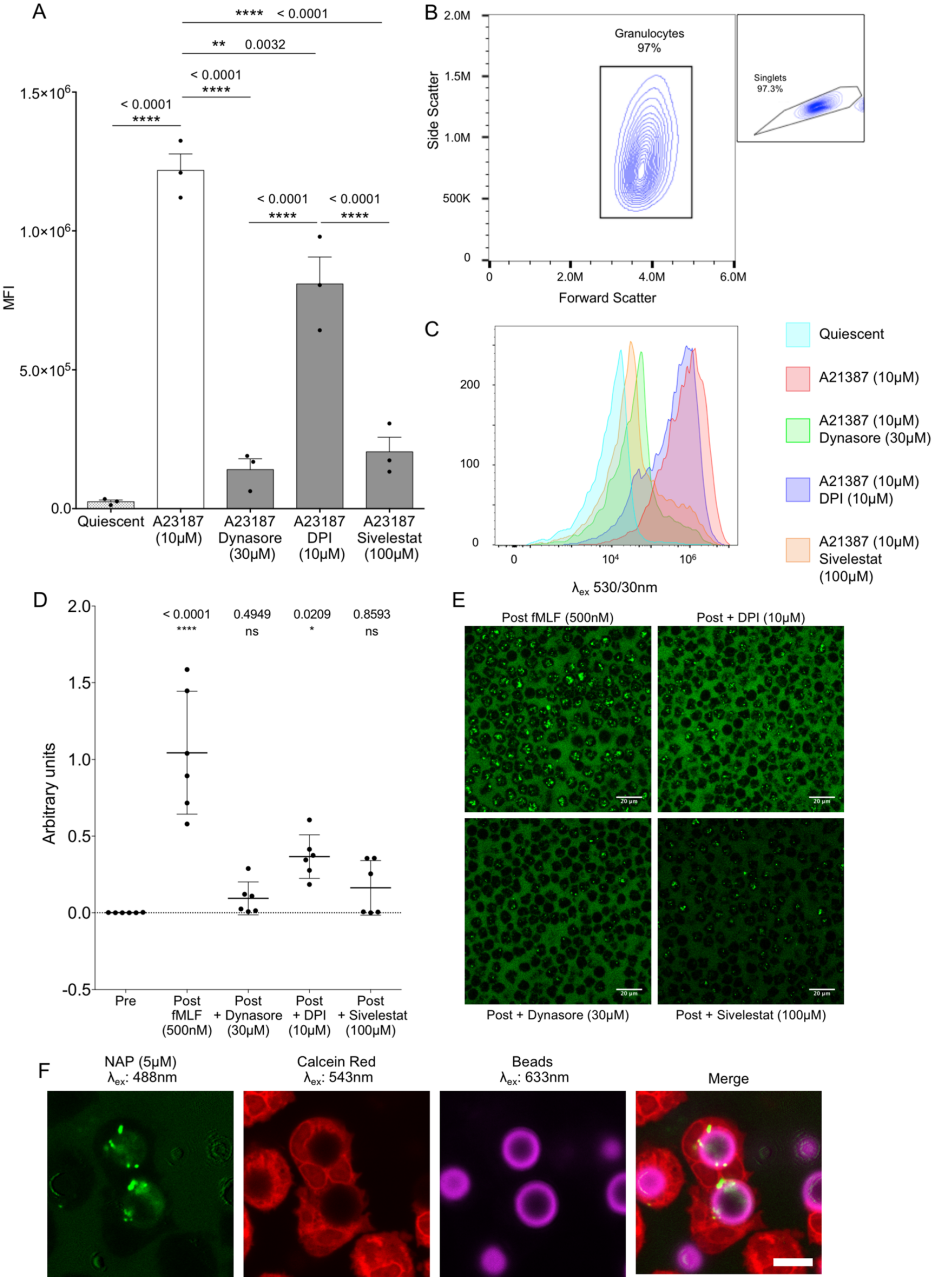
**(A)** Mean fluorescence index of entire granulocyte population as determined by flow cytometry. Quiescent (spotted bar), activated (white bar) and inhibited (grey bars) conditions are shown. Plots and images are representative from three independent experiments. Graph shows mean (± SEM) from three independent experiments. Statistical analysis by one-way ANOVA with Bonferroni’s correction, F = 73.80, DF = 12, exact multiplicity adjusted P value for each comparison shown in the graph. **(B)** Example flow plot of freshly isolated healthy human PMNs. Target population and percentage of parent gate shown (inset: parent gate). **(C) **Example histograms of de-quenched intracellular NAP fluorescence amongst forwarded PMNs are shown with key. The addition of each inhibitor partially returns the fluorescent intensity back towards the quiescent baseline. Plots are representative of three independent experiments. **(D)** Bar chart indicating the cellular fluorescence determined by objective image analysis in repeated field views across the incubation conditions. Results are mean (± SD). Statistical analyses determined by one-way ANOVA with post-hoc Bonferroni correction. F = 23.55, degrees of freedom 25, exact multiplicity corrected p value for each comparison shown. *ns* not significant, * p < 0.05, ** p < 0.01, *** p < 0.001, **** p < 0.0001, Three fields of view from two independent experiments. **(E)** Example images of isolated neutrophils, primed with Cytochalasin B (10.4 µM), exposed to fMLF (500 nM) immediately after t = 0 s in the continued presence or absence of the various inhibitors as shown. **(F)** Fluorescent beads were opsonized and incubated with isolated healthy neutrophils in the presence of NAP. De-quenched green fluorescent signal co-localizes with the beads indicating the origin of intense fluorescence to be the phagosome. Image is representative of two independent experiments. Scale bar 5 µm.

Intracellular fluorescent green signal was quantified using flow cytometry. In the presence of A23187 (10 µM) there was a 50-fold increase in intracellular fluorescent signal amongst freshly isolated neutrophils (Fig. 3A; gating strategy shown in Fig. 3B) as compared to the threefold increased seen with free human neutrophil elastase (Fig. 1C). The intracellular fluorescent signal could be abrogated by concurrent dynamin inhibition (Dynasore Hydate), which inhibits pinocytosis. NADPH oxidase inhibition (Diphenyleneiodonium, DPI) reduced the overall mean fluorescent intensity in keeping with the role of NADPH oxidase contributing to the progressive alkalinisation of the neutrophil vacuole following phagocytosis. The intracellular signal was also abrogated by co-culture with Sivelestat indicating a contribution from elastase activity to the intracellular fluorescent signal (Fig. 3A,C). The contribution of these processes to the liberation of fluorescent signal was confirmed using confocal microscopy, with Cytochalasin B (10.4 µM) and fMLF (500 nM) used to activate neutrophils (Fig. 3D,E). When neutrophils phagocytosed opsonized fluorescent beads in the presence of NAP, the de-quenched fluorescent signal co-localized with the beads (Fig. 3F). Additionally, when neutrophils phagocytosed fluorescently labelled *Pseudomonas* *aeruginosa,* the de-quenched signal co-localled with the bacteria (Supplemental fig. S4). Both these results indicate that intracellular probe dequenching occurred within the phagosome.

### NAP manufactured to GMP standard was chemically stable and non-toxic

Having demonstrated that locally delivered microdoses of NAP coupled with OEM could detect activated neutrophils in vitro in situ within minutes, we manufactured NAP to GMP standards and performed preclinical toxicology and chemical stability analysis. NAP did not cause red cell haemolysis or neutrophil necrosis (Supplemental Fig. S5). NAP was delivered via the trachea to rats in a Contract Research Organization Good Laboratory Practice (GLP) toxicology study at approximately 700 times the human dose. NAP (100 µg) was instilled and rats underwent necroscopy at 3 and 15 days after delivery. No local or distant organ toxicity was observed and there were no haematological or other metabolic consequences (Covance Laboratories, Harrogate, UK). A GLP AMES mutagenicity test using the microsuspension-modified method appropriate for early clinical development demonstrated no mutagenicity. Stability assessment (Department of Chemistry, University of Edinburgh) demonstrated that NAP drug substance (as a dry powder) was stable for up to 4 years and 8 months at – 20 °C and as drug product (in aqueous solution) was stable for up to 2 years when kept at room temperature (Supplementary Tables S1 and S2). The release specifications for NAP drug substance and drug product are shown in Supplementary tables S3 and S4.

### NAP permitted visualization of activated neutrophils in the alveoli in patients in an exploratory clinical study

**Figure 4:**
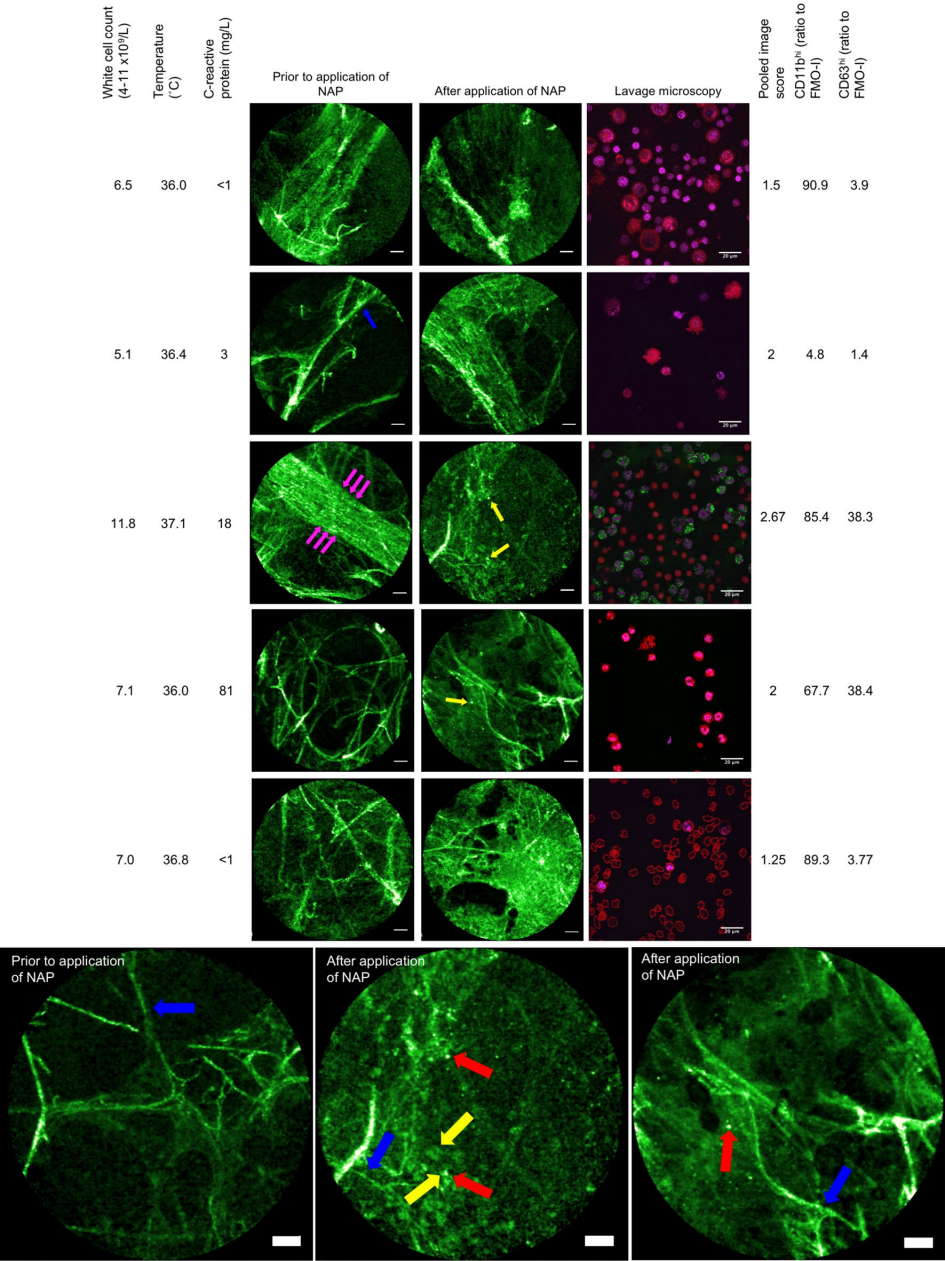
Representative OEM images from participants with bronchiectasis before and after the application of NAP are shown in the left-hand image columns. One participant received NAP but did not tolerate subsequent imaging, so five sets of imaging are shown. Blue arrows—alveolar wall, red arrow—punctate fluorescent NAP signal, purple arrows—pulmonary capillary. The labelling of relevant features is not exhaustive in these representative images. Laboratory confocal microscopy from the broncho-alveolar lavage is shown with each pair of OEM images. Red stain—Calcein red cytoplasmic stain. Purple stain—Styo62 nuclear stain. Green stain—material excited at 488 nm and emitting at 500–530 nm. Calcein red and Syto62 are applied after lavage harvest. Green fluorescent staining has taken place within the lung. Left hand side: selected clinical parameters from each participant are shown. Right hand side: pooled subjective blinded video analysis from two blinded experienced observers working independently is shown. 1—no suspicion of NAP signal, 2—suspicion of NAP signal, 3—positive NAP signal, 4—very positive NAP signal. Of the CD66b^hi^ population in lavage, values for CD11b^hi^ and CD63^hi^ cell surface markers as a ratio of the relevant isotype control are shown. Bottom row; larger example OEM images for inspection, description given with image. Scale bar OEM 50 µm, scale bar laboratory confocal microscopy 50 µm.

Six healthy volunteers, six patients with bronchiectasis and three mechanically-ventilated critically ill patients completed the study. There were no adverse reactions related to NAP administration (Supplementary Table S5). As expected there were no increases in punctate cellular signal seen after the intrapulmonary administration of NAP in the healthy volunteers (Supplementary Fig. S6). Amongst bronchiectatic patients a range of fluorescent responses following the application of NAP were observed. Investigation of these patients provided the opportunity to obtain BALF after performing OEM with NAP. Using flow cytometry neutrophil activation status was inferred by measuring CD63 (membrane protein associated primary neutrophil granules^25^) and CD11b (cell surface marker of adhesion associated with the process of activation). Variable cell surface marker up regulation was detected (Fig. 4). Laboratory confocal microscopy was also performed on lavage directly following nuclear staining, and in one case where neutrophils were CD11b/CD63 high, neutrophils containing green punctate fluorescent were observed. Two blinded adjudicators displayed fair concordance when using a four-point ordinal scale to provide a subjective assessment of 26 separate OEM/NAP video sequences taken across the 11 study participants who received the full dose of NAP (Cohen’s Kappa 0.247 ± SE 0.101), and their scores are shown in Fig. 4. Example video sequences can be viewed with Supplementary videos S2 and S3.

**Figure 5:**
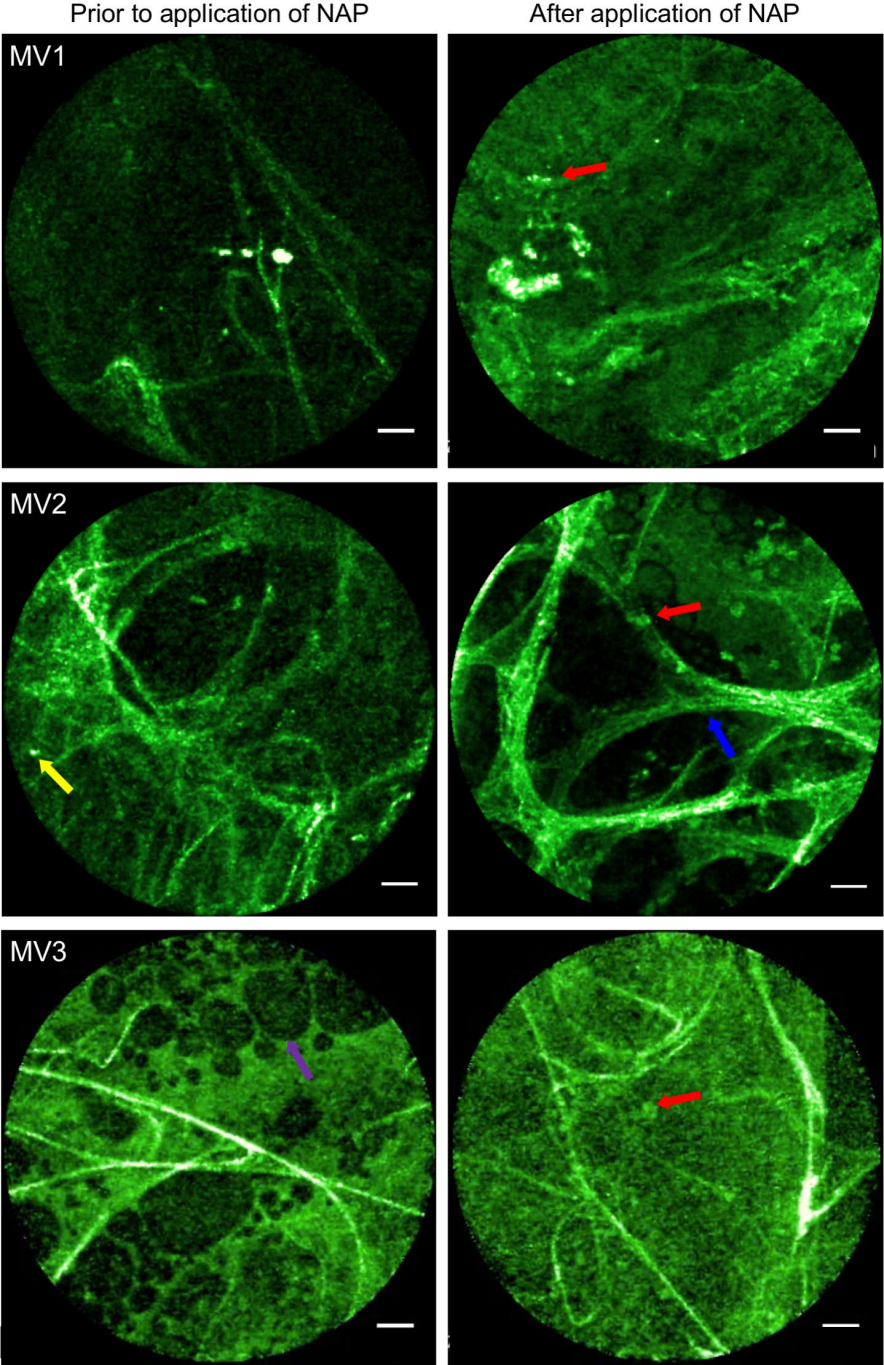
OEM from three mechanically ventilated (MV) patients. Each pair of images are representative from an individual study participant. Blue arrows—alveolar wall, red arrow—punctate fluorescent NAP signal, yellow arrow—autofluorescent cell, purple arrow—bubble of fluorescent fluid. The labelling of relevant features is not exhaustive in these representative images.

We then demonstrated that the procedure is feasible in MV patients in the complex and demanding environment of an intensive care unit (ICU). Still images before and after the administration of NAP in three MV patients are shown in Fig. 5. Some cells with intracellular punctate fluorescent signal are seen after the application of NAP (red arrows). However, punctate signal was seen prior to the application of NAP indicating the presence of auto-fluorescent cells undermining the discriminatory value of NAP unless comparison to baseline is made. In one participant auto-fluorescent fluid was observed (purple arrows) attributable to bilirubin which is known to fluoresce at this wavelength and this individual was jaundiced.

## Discussion

Neutrophils are the first line of defense in the innate immune system. Neutrophil activation and response includes a wide range of orchestrated events including: migration to site of injury, rapid phagocytosis, release of destructive enzymes, and production of reactive oxygen species. Whilst these pathways bring about pathogen destruction, neutrophil activation is also implicated in the pathogenesis of many acute human diseases. Most of these phenotypic changes are dynamic and are not suitable for external static assessment following sample removal. Pathological evaluation of biopsied tissue aids the classification of diffuse inflammatory parenchymal lung diseases. However, the lack of repeatability, small biopsy volume, and the static nature of the samples are potential limitations which support the development of more dynamic approaches to augment pathological sampling.

In contrast, real-time in situ characterization presents an opportunity to understand the contribution of neutrophil activation in patients with disease and stratify patients according to biological process rather than clinical phenotype. There is emerging evidence that neutrophil activation and migration from the circulation occur simultaneously^26–28^, but this has not been characterized in acutely unwell patients or exploited for clinical purposes. To date, in vivo neutrophil-specific molecular imaging has been limited to extraneously labeled neutrophils^29^ or the use of [^18^F]-fluorodeoxyglucose as a surrogate of inflammatory cell metabolic burst activity^27,28,30^. Optical imaging has been used extensively for pre-clinical imaging of various aspects of neutrophil activation with a particular focus on enzymatic imaging, but no technique has been deployed for clinical use.

We developed an activated-neutrophil phenotype-specific imaging agent for application directly into human lungs for read out using optical endomicroscopy. We exploited three biochemical processes unique to activated neutrophils namely dynamin dependent endocytosis, phagosome alkalinization and elastase release and chemically modified a previously reported compound^23^, with the intention of translating into human disease via endomicroscopy. In its resting state the three fluorescein moieties are quenched internally. The multibranched scaffold exploits the propensity of professional phagocytes to endocytose large molecules^31^, a process dependent upon the action of dynamin, a GTPase that separates newly formed vesicles from the cellular membrane^32^. Whilst dynamin is ubiquitous to eukaryotic cells, relying on dynamin-dependent endocytosis to rapidly concentrate NAP in the intracellular environment is the first step in targeting neutrophil activation. Unlike other professional phagocytes, where acidification of the phagosome is used as a mechanism for the destruction of ingested material, the neutrophil vacuole undergoes initial alkalinization^33^. This process is brought about by the activity of the vacuolar membrane protein NADPH oxidase, which facilitates depolarization of the vacuolar membrane, flooding the vacuole with electrons used in the generation of oxygen super-radicals^34^. Protons are consumed through dismutation of radicals and the pH rises^35^. This pH rise is more than just an unintended sequitur of the production of reactive oxygen species; it plays a role in the activity of HNE. HNE is normally sequestered in primary granules within the cytoplasm of quiescent neutrophils. It is released in the extracellular milieu and into phagosomes containing ingested material^36,37^. It has a higher activity at pH 9 than at neutral pH, making it more destructive within the phagosome, and less destructive outside, limiting damage to host tissue^38^. Segal first demonstrated the alkalization of the neutrophil vacuole by using fluorescein conjugated to *Staphylococcus* *aureus*^33^*,* and thus we use the same pH sensitive fluorophore on our SmartProbe. Consequently, we observe a partial abrogation of fluorescent signal when alkalization of the vacuole is inhibited, rather than complete abrogation, because fluorescein is still fluorescent at neutral pH. Each fluorescein moiety on our SmartProbe is connected through a polyethylene glycol chain to the amino-acid sequence EEINleRR. In the presence of HNE, the sequence is hydrolyzed, internal quenching is liberated and fluorescence increases. Through selective inhibition of these three processes, we demonstrate that NAP identifies an activated neutrophil phenotype according to the postulated mechanisms. The three pathways that we investigated as contributors (pinocytosis, alkalinisation, elastase) to probe activation in vitro, may be further dissected by dose–response experiments and/or by using different combinations of NADPH oxidase inhibitors and ROS scavengers or neutrophils from patients with chronic granulomatous disease (CGD). However, it is highly likely that these pathways are inseparable in the clinical environment and are unlikely to correlate with the clinical findings. The peptide sequence confers the ability of the SmartProbe to act as HNE sensor in isolation but in this capacity the performance is indeed limited. The signal-to-noise ratio of the SmartProbe is low in the extracellular environment where dequenching only occurs due to action of HNE, of the order of 3 to 1, and takes many minutes to reach maximal fluorescence. Existing HNE probes exhibit similar or superior performance in this regard^18,39^ and have been deployed for in vivo fluorescent surface imaging of HNE activity, albeit limited to small animal work. However, in the complex intracellular environment the signal of our probe is very bright, with a signal-to-noise ratio of approximately 50 to 1, with peak fluorescence generated within two minutes of neutrophil activation, and thus an ideal profile to identify activated neutrophils after the local delivery of a SmartProbe into the distal lung.

So far, few molecular imaging strategies have been translated to human studies. Hurdles of cost of development, cumbersome equipment, and the use of ionising radiation have hindered development of nuclear modalities such as positron-emission tomography (PET). Optical molecular imaging employs wavelengths of the electromagnetic spectrum that do not require ionising radiation and are relatively low cost^40^. Visible wavelengths of light exhibit a lack of tissue penetration (at most a few millimetres), which has been a limitation for human organ imaging, but the use of fibre-based approaches, such as OEM, may represent a solution by allowing microscopy of any endoscopically-accessible organ system. We recently demonstrated the capability of the optical imaging/SmartProbe strategy to detect bacteria in the lungs of critically ill patients, with applicability to patients with severe community acquired and ventilator-associated pneumonia^19^. In order to proceed to human study, we adopted a microdosing approach, whereby each single application of an active pharmaceutical ingredient does not exceed 100 µg, with the inherent safety advantages this provides. NAP was non-toxic and chemically robust with over four years of stability demonstrated for the dry drug substance and two years for the aqueous drug product (drug substance dissolved in phosphate buffered saline). After completing a Phase 1 healthy volunteer study, we demonstrated the punctate signal of activated neutrophils in a subset of patients with bronchiectasis, giving imaging characteristics similar to those observed in vitro. Even amongst a small number of patients chronically unwell with bronchiectasis, we observed heterogeneity of neutrophil activity. There was no obvious routinely available clinical information that predicted the occurrence of a positive NAP signal, strengthening the case for the development of such translational tools in clinical practice in order to interrogate patient-specific biological processes in ways not available with traditional systemic assessments. The demonstrated coupling of bespoke optical molecular imaging probes (SmartProbes) and OEM can readily be added to standard bronchoscopic assessments, making the approach particularly suitable in the surveillance and diagnosis of patients with acute lung inflammation requiring MV, and so we progressed to demonstrate the safety and feasibility of such an approach in three MV patients.

There are some acknowledged limitations to the current work. The imaging agent is designed to optimally absorb light of 480 nm wavelength and emit above 520 nm, a design implicit upon the single-wavelength OEM devices that are clinically certified for use at this time. The use of fluorescein is a pre-requisite at this stage, due to the pH-dependent fluorescence and the vast historical safety record in human application. This leads to significant overlap with endogenous green autofluorescent signal from connective tissue in the lung and potential challenges to confidently identify the optical signal in the complex in vivo microenvironment. In addition, there is a lack of robust objective image analysis hampered in part by the complex nature of image that is constructed, the lack of immediately discriminant features such as colour, and a lack of data on which to train an objective analysis algorithm. Other limitations are more fundamental: in comparison to routine laboratory assessments, the varied origin of probe dequenching and the variable dose that may reach the alveolus could be criticized for lacking precision, but the lungs of sick patients are not fully represented by the controlled samples of the laboratory. Here, some precision has necessarily been pragmatically sacrificed in order to interrogate a biological process that has not previously been documented in vivo in situ in humans. At this stage of development, our current work does not demonstrate the utility of this imaging technique for patient stratification. For example, in the critically ill, stratification of patients by alveolar neutrophil activation is hypothetical, due to the current poor understanding of in vivo in situ pathology in humans and the consequent lack of proven biological therapies. Indeed, critically ill patients displaying a heterogeneity of diseases and disease severities are difficult to study, and there are currently few acceptable disease reference standards. However, the very lack of these reference standards supports the development of novel methodologies to specifically incorporate biological process into the diagnosis and stratification of disease.

In summary, we report the development and initial experience of an optical molecular imaging approach capable of rapidly identifying activated neutrophils in the human alveolar space that can be applied at the bedside and yield immediate biological data. In a Phase 0/1 clinical trial of the investigational medicinal product, we demonstrated that NAP coupled with OEM is safe, well tolerated, shows promising imaging characteristics, and is the first demonstration of in vivo microscopic imaging of neutrophil activity in humans with lung disease. No firm conclusions can yet be drawn regarding the ultimate utility of the technique. To that end, a phase 2 study has begun to evaluate the reliability and validity of this novel technique to quantify neutrophil activation, predict outcome and stratify MV patients in the intensive care unit. (Sensing using the Neutrophil Activation Probe on the Intensive Therapy unit, SNAP-IT, NCT: 02804854 EudraCT: 2015-005676-25).

## Methods

### Ethics statement

All experiments using human volunteers were performed following approval from the appropriate regional ethics committee (REC) and informed consent was obtained from each participant or their personal legal representative for adults with incapacity (in accordance with the Adults with Incapacity (Scotland) Act 2000). BALF for alveolar macrophages: (East of Scotland Research Ethics Committee 1 no: 07/S1102/20), blood for assessment of leucocyte populations (Lothian Research Ethics Committee no: 08/S1103/38), and first-in-human assessment of probe performance and safety (Scotland A Research Ethics Committee no: 12/SS/0004, EudraCT 2011-006169-17, NCT01532024). All methods were performed in accordance with the relevant guidelines and regulations. Animal toxicology was conducted in compliance with UK Good Laboratory Practice Regulations by a third party contract research organization (Covance Laboratories Ltd, Harrogate, UK) under Covance UK Home Office License for toxicology. All procedures were approved by and performed in accordance with Institutional Committees on Animal Welfare of the UK Home Office in compliance with The Home Office Animals Scientific Procedures Act, 1986 (Project licence number 70/7602-1). All procedures were carried out by a personal licence holder.

### Chemical synthesis of NAP

**Figure 6:**
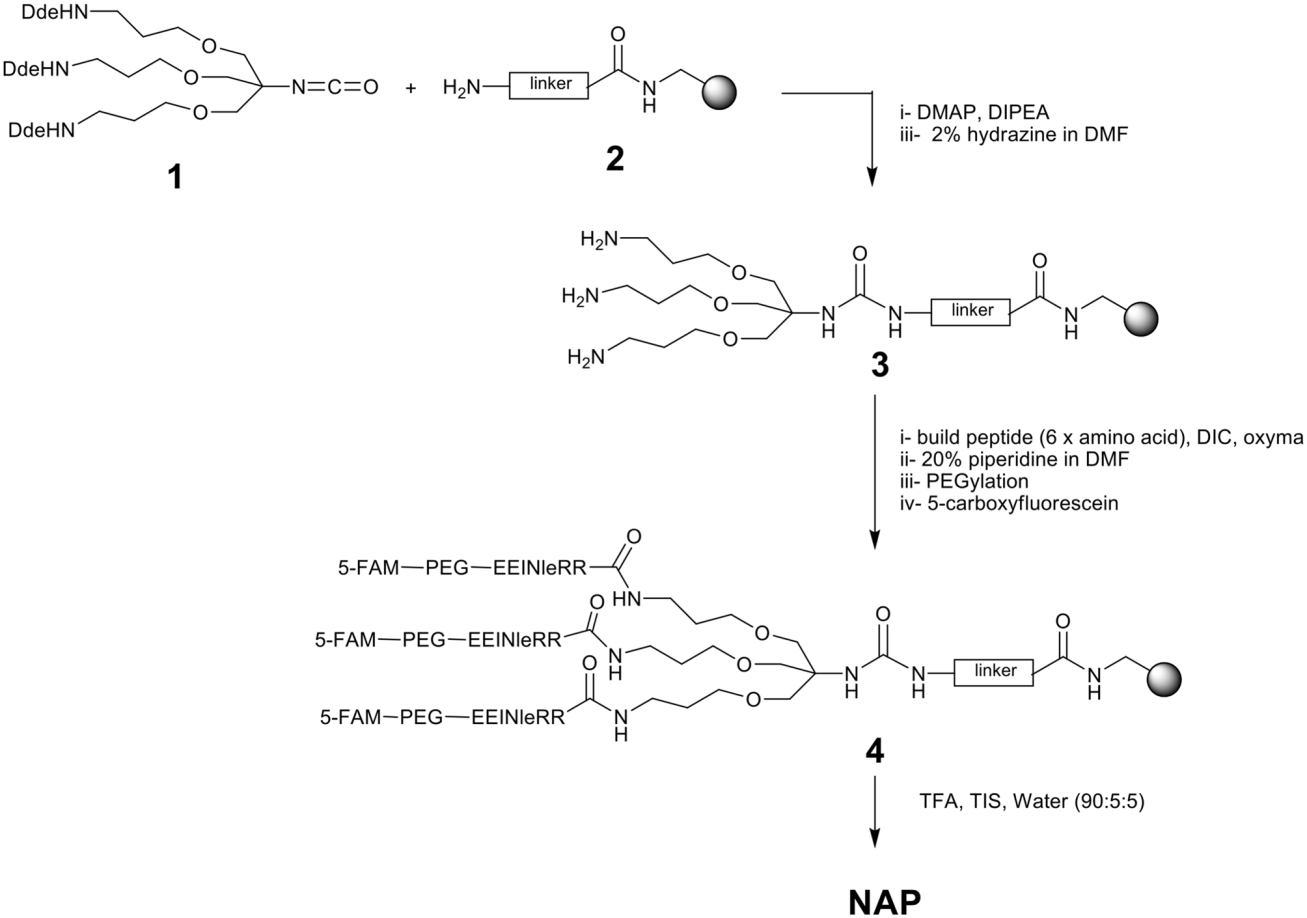
Synthesis of NAP.

The synthetic route to manufacture NAP is shown in Fig. 6. The dendrimer scaffold **1**^23^ is linked to a solid support to permit further conjugation. The peptide sequence (–Glu–Glu–Ile–Nle–Arg–Arg–) is built and a solubilizing moiety (PEG) is added along with the fluorescent dye, carboxyfluorescein. NAP is then released from the solid support followed by a purification step.

### Synthesis of resin-bound dendrimeric scaffold (3)

Resin **2** was synthesized using a 4-[(2,4-dimethoxyphenyl)-(Fmoc-amino)methyl]phenoxyacetic acid (Rink amide linker) attached to aminomethyl PS resin (1% DVB, 100–200 mesh, 1.43 mmol/g capacity). Thus Fmoc-Rink-amide linker (3 eq) was dissolved in DMF and Oxyma (3 eq) was added and the mixture was stirred for 10 min. DIC (3 eq) was then added and the resulting mixture was stirred for further 5 min. The solution was added to aminomethyl polystyrene resin (1 g, 1.43 mmol/g) and shaken for 2 h. The resulting resin was washed with DMF (3×), DCM (3×) and MeOH (3×).

### Fmoc—deprotection

To the resin (pre-swollen in DCM) was added 20% piperidine in DMF and the reaction mixture was shaken for 10 min. The solution was then drained, and the resin was washed with DMF (3×), DCM (3×) and MeOH (3×). This procedure repeated twice.

### Isocyanate coupling

To resin **2** (1 g, 1 eq), pre-swollen in DCM (10 mL), was added a solution of isocyanate monomer^23^ **1** (5 g, 3.0 eq), DIPEA (3.0 eq) and DMAP (0.6 eq) in a mixture of DCM/DMF (1:1, 20 mL) and the mixture was shaken for 16 h and the reaction monitored by a quantitative ninhydrin test^41^. The solution was drained, and the resin was washed with DMF (3×), DCM (3×) and MeOH (3×).

### Dde—deprotection

To the resin (1 g), pre-swollen in DCM, was added 2% hydrazine in DMF (20 mL) and the reaction mixture was shaken for 2 h. The solution was then drained and the resin **3** was washed with DMF (3 × 20 mL), DCM (3 × 20 mL) and MeOH (3 × 20 mL).

### Peptide, PEG and dye coupling for dendrimer (4)

A solution of the appropriate Fmoc-amino acid (10 eq) (Fmoc-Arg(Pbf)-OH, Fmoc-Nle-OH, Fmoc-Ile-OH, Fmoc-Glu(tBu)-OH, Fmoc-PEG-OH, 5-carboxyfluorescein) and Oxyma (10 eq) in DMF was stirred for 10 min (with 3 eq of acid per amine at 0.1 M concentration). DIC (10 eq) was then added and the resulting solution was stirred for further 5 min. The solution was then added to resin **3** (1 eq), pre-swollen in DCM, and the reaction mixture was shaken for 0.5 h. The solution was drained, and the resin was washed with DMF (× 3), DCM (× 3) and MeOH (× 3). The coupling reactions were monitored by a quantitative ninhydrin test^41^. Before the final cleavage, the resin was washed with 20% piperidine to remove any fluorescein phenol esters^42^.

### TFA cleavage and purification of NAP

The resin **4** (initial weight 1.5 g), pre-swollen in DCM, was treated with a cleavage cocktail of TFA/TIS/water (90/5/5) for 2 h at room temperature. The solution was drained and the resin was washed with 50% TFA/DCM solution. The peptide was precipitated by ether. The precipitated solid was collected by centrifugation and the solvent removed by decantation and the precipitate was washed with cold ether (3×). The peptide was dried in vacuum at ambient temperature for 6 h to give crude output of 5.7 g. The precipitate was then purified by reverse phase preparative HPLC and the required fractions were pooled and lyophilized to afford NAP as an orange odorless powder (1.4 g). HPLC (220 nm): *t*_*R*_ = 3.4 min, purity > 99%; Res. Sol: acetonitrile not detected by GC–MS; MALDI: C197H277N44O61: [M + H^+^] calculated: 4236.88, [M + H^+^] found: 4236.02.

### Plate reader assessment of probe de-quenching

To generate an emission spectrum, NAP was excited at 430 nm to ensure the spectrum could be visualized to the x-axis. Excitation/absorbance spectra were obtained with NAP diluted to 5 µM in PBS or freshly prepared pH buffer. Purified HNE (Elastin Products Company, MO, USA) and purified proteinase 3 (Sigma-Aldrich, Poole, UK) were made up according to the manufacturer’s instructions to yield appropriate stock concentrations. Stock preparations of purified enzyme were prepared according to manufacturers’ instructions and subsequently diluted appropriately with Phosphate Buffered Saline (PBS, Thermoscientific, MA, USA) and NAP to achieve a final volume of 200 µL per condition and a final NAP concentration of 5 µM. Solutions were prepared directly into 96 well plates (Corning, NY, USA) on ice, with or without the human neutrophil elastase inhibitor Sivelestat (ONO-5046, 100 µM, Tocris, Bristol, UK). Plates were subsequently incubated at 37 °C with fluorescent parameters recorded every minute for 1 h (Fig. 1C, excitation 488 nm, emission 525 nm). All fluorescence parameters were recorded using a microplate reader (Synergy H1 hybrid reader, BioTek, Potton, UK,).

### Leucocyte isolation

Whole blood was obtained from peripheral venepuncture of healthy volunteers using a 19-gauge hollow needle and anticoagulated immediately. Polymorphonuclear cells (PMNs) and peripheral blood mononuclear cells (PBMCs) were isolated as previously described^43^. For mixed leucocyte populations the neutrophil isolation procedure was arrested after dextran sedimentation of erythrocytes before the separation of PMNs and PBMCs. Leucocytes were suspended at the concentrations specified below in PBS with divalent cations for 30 min prior to any further experimentation. All cell counting was performed using an automated cell counter (NucleoCounter® NC-100™, ChemoMetec, Allerød, Denmark).

### Lavage preparation

Following broncho-alveolar lavage samples were passed through to a 40 µM cell strainer to eliminate mucus and other debris. Cells were centrifuged as previously described and reconstituted in PBS with Ca^2+^/Mg^2+^ at a concentration of 1 × 10^6^/mL. The supernatant underwent fluorescent plate reader analysis to confirm that lavage had been taken from segments also exposed to NAP (data not shown).

### Confocal imaging

Where stated commercial stains were used in accordance with the manufacturer’s instructions or applied to a suspension of the relevant cells in eppendorfs at a concentration of 1:1000 from delivered stock for 10 min at 37 °C and 500 rpm using an orbital plate shaker before undergoing one wash in PBS without divalent cations as described above. 8 well Lab-Tek II confocal chambers (VWR, PA, USA) were coated in fibronectin (1 µg/ml, Sigma-Aldrich) for 10 min at 37 °C. Wells were then washed twice before the addition of the appropriate cell suspension. Cells were typically seeded at a concentration of 0.5—1 × 10^6^/mL. Cells were allowed to seed for 20 min at 37 °C after which the remaining supernatant was washed once using PBS without divalent cations. Where used, 200 µL of NAP (5 µM) was then applied to each well and imaging proceeded. For time course experiments PMNs were exposed to Cytochalasin B (10.4 µM) during seeding and fMLF (500 nM) after the initiation of time course imaging. Fluorescent beads (Micro particles based on melamine resin, carboxylate-modified, nile blue-marked size: 6 μm, Sigma-Aldrich) were opsonised before being added directly to adherent neutrophils. *Pseudomonas* *aeruginosa*, at one optical density, were labelled with PKH (CellVue claret far red fluorescent cell linker kits, Sigma-Aldrich) according to the manufacturer’s instructions and added to adherent neutrophils. Incubation proceeded for 30 min at 37 °C prior to the addition of Calcein-AM red–orange cytoplasmic dye (500 nM, Life technologies). Following a single wash with PBS, NAP was added to a final concentration of 5 µM. Imaging proceeded within 10 min.

A laser scanning confocal imaging system (LSM510; Carl Zeiss, Jena, Germany), incorporating an upright Axioskop FS2 microscope (× 63 objective) was used for image acquisition. 488 nm laser power was limited to 5% of maximum to minimize phototoxicity. Fluorescein based dyes were excited with a dedicated 488 nm laser line, with emitted light detected at 500–530 nm. Syto 82 nuclear, calcein cytoplasmic dyes and CellMask Orange plasma membrane stain (all Invitrogen, Thermoscientific) were excited with a dedicated 543 nm line, with emitted light detected at 560–600 nm. Syto 60 (Invitrogen, Thermoscientific) stain was excited with a dedicated 633 nm line, with emitted light detected at 650–680 nm. PKH labelled bacteria were excited with a dedicated 633 nm line with emitted light detected at 650–705 nm. Detector gain and offset were adjusted with each use to achieve the highest dynamic range with smallest saturation.

### Image analysis of laboratory confocal images

Image analysis was carried out using Fiji (version 2.0.0). An algorithm was developed to calculate the area of each image taken up by intracellular punctate fluorescence as a fraction of the area of the image taken up by cells. The Trainable Weka Segmentation plugin (v3.1.0) was used to differentiate cells from background. To calculate the numerator each image was filtered using a default threshold to eliminate all but intracellular fluorescent signal. Particle analysis of the resulting binary mask was used to calculate the appropriate number of pixels. Isolated healthy quiescent neutrophils from each experimental replication were taken as a baseline, and quantification of fluorescent signal is expressed in arbitrary units as a ratio of the corresponding baseline.

### Flow cytometric evaluation

Neutrophils were suspended in PBS with divalent cations at a concentration of 10 × 10^6^/mL. 0.85 × 10^6^ /mL neutrophils were incubated in a final volume of 100 µL in 2 mL microtubes (Eppendorf, Hamburg, Germany) with or without the following pharmacological agents: A23187 (Calcium Ionophore, 10 µM, Sigma-Aldrich), Dynasore Hydrate (30 µM, Sigma-Aldrich), Diphenyleneiodonium (DPI, 10 µM, Sigma-Aldrich), Sivelestat (100 µM, Tocris). Incubation took place at 37 °C for 30 min and samples were shaken at 500 rpm using an orbital plate shaker (Thermomixer comfort, Eppendorf, Hamburg, Germany). Suspensions were then incubated with anti-human CD11b-APC (1:100 dilution, Clone ICRF44, Biolegend, CA, USA) at 22 °C for 30 min at 500 rpm before the application of NAP to a final concentration of 5 µM for 15 min at 22 °C and 500 rpm. Cells were washed in PBS without divalent cations at 350 g for 5 min at room temperature using a microtube centrifuge (Biofuge fresco, Heraeus, Hanau, Germany) and reconstituted in 175 µL PBS without divalent cations. Samples were loaded onto a 96 round-bottomed well plate (Corning).

For the demonstration of NAP specificity to PMNs, a mixed leucocyte population was prepared as above and suspended in PBS with divalent cations at a cellular concentration of 10 × 10^6^/mL. Where appropriate, samples were activated with A23187 (Calcium Ionophore, 1 µM) for 30 min at 37 °C and 500 rpm using an orbital plate shaker. 100 µL of prepared suspension was mixed with 100 µL of flow cytometry staining buffer solution (eBioscience, CA, USA) and then samples were incubated for 30 min at 22 °C and 500 rpm in the presence of anti-human CD45-PE (1:100 dilution, Clone HI30, Becton–Dickinson) and anti-human CD66b-AF647 (1:100 dilution, Clone G10F5, Becton–Dickinson). NAP was then added to achieve a final concentration of 5 µM and microtubes were incubated at 22 °C and 500 rpm for a further 15 min. Samples were washed, reconstituted and loaded as above. The flow cytometry gating strategy shown in Supplementary Fig. S3B.

For the assessment of lavage samples, 100 µL of cell suspension was mixed with 100 µL of flow staining buffer (eBioscience, 00–4222-26) and CD66b-PE (1:50 dilution, Clone G10F5, Becton–Dickinson), CD63-PE/Cy7 (1:50 dilution, Clone H5C6, Becton–Dickinson), CD11b-APC (1:50 dilution). Fluorescence minus one plus isotype (FMO-I) controls were included for each fluorescent parameter. Cell suspensions were incubated at 4 °C for 30 min using an orbital plate shaker. Cells were washed once into PBS without cations. Lavage PMNs were identified by the CD66^hi^ status. Fluorescent intensity from each compensated parameter was expressed as a function of the relevant FMO-I control for each patient (Supplementary Fig. S7).

Flow cytometry was performed using an Accuri C6 flow cytometer and BD CSampler software (both Becton–Dickinson, NJ, USA). Compensation beads and single stain controls were used where appropriate. On one occasion, due to technical malfunction, a BD Calibur flow cytometer using BD CellQuest Pro software was used to analyse lavage samples. The cytometer was thresholded on forward scatter to eliminate debris. The cytometer was aligned monthly using Calibrite 3, APC and 8 peak beads (Becton–Dickinson) to calibrate scatter and fluorescence parameters.

Data analyses were performed using Flowjo v10.0.8r1 (Treestar, Ashland, OR, USA). Samples were repeated in duplicate for each individual donor and pooled to yield a point estimate for each condition and each donor. The point estimates from different donors were pooled to yield an overall mean point estimate for each condition ± SE.

### Cellular toxicity

Membrane toxicity of NAP was assessed using erythrocyte haemolysis. Isolated erythrocytes remaining after leukocyte separation were diluted 1:5 with PBS without cations and 50 µL of this red cell suspension was added to wells of a 96-well plate. Working concentration of NAP (5 µM), or working concentration × 2, was added to the relevant well. Positive control wells contained Triton X-100 (0.4%, Sigma-Aldrich) and negative control wells contained PBS w/o only. The 96-well plate was incubated for one hour at 37 °C in a humidified environment. Following incubation, 180 µL of PBS without cations was added to each well and the plate was centrifuged at 2500 rpm for 10 min. 100 µL of supernatant from each well was removed and aliquoted into clean wells. Absorbance at 350 nm was recorded using a plate reader. Triplicate repeats were pooled to yield an overall mean point estimate ± SEM of haemolysis that was expressed as a percentage of the positive control. For neutrophil toxicity freshly isolated human peripheral neutrophils were resuspended at 10 × 10^6^ cells/mL in IMDM with 10% autologous serum and cultured for 18hrs ± 5 µM NAP at 37 degrees in a 5% CO_2_ incubator. As a positive control for death, neutrophils were snap frozen on dry ice prior to thawing in hot water. Neutrophils were dislodged via pipetting, and 40 µL transferred into flow tubes containing Annexin V (1:1000) in Annexin V binding buffer (HBSS + calcium + magnesium containing 5 mM Calcium chloride) and incubated on ice for 10 min. Immediately prior to flow cytometry (5L Fortessa, BD) 1 µL propidium iodide (from 100ug/mL stock) was added. Duplicate repeats were pooled to yield an overall mean point estimate ± SEM of apoptosis and necrosis that was expressed as a percentage of total cells.

### Optical endomicroscopy

A commercially available 488 nm excitation CLE platform and 1.4 mm diameter tip optical fibre was utilized (Cellvizio 488 with alveoflex fibre, Mauna Kea Technologies, Paris, France) along with the associated proprietary software (Image cell v3.8, Mauna Kea Technologies). Alveoscopy videos were acquired at 12 frames per second. For in-human investigations a clinically approved version of the Cellvizio 488 laser-scanning unit (with associated clinically rated proprietary software) was used. Videos and still images were prepared for presentation using Cellvizio Viewer 1.6.2 (Mauna Kea Technologies). Videos were automatically converted to gray-scale by the viewer software, so were false colored green-white to illustrate that the wavelengths being detected were predominantly in the green region of the visible spectrum. Where applicable, if two or more videos or stills were visually compared, the automatic software performing continuous auto-gain for optical dynamic range was disabled and gain was fixed.

### Exploratory clinical study

The study was registered with the EudraCT database (EudraCT number: 2011-066167-17) and with the National Clinical Trials database (NCT01532024). The primary objective was feasibility: could activated neutrophils within the alveolar space be detected with NAP and OEM over background autofluorescence***.*** The secondary objective was safety: defined by the occurrence of adverse events. Six healthy male volunteers were recruited through study specific advertisements distributed via University of Edinburgh mailing lists. Participants gave written acknowledgement of informed consent after receiving oral and written explanation of the study. Healthy volunteers attended the Edinburgh Clinical Research Facility within the Royal Infirmary of Edinburgh for all study specific events. After consent was provided potential participants were checked against the over-volunteering prevention system before undergoing medical screening. Participants’ general practitioners were asked to confirm the past medical history before volunteers were approved to participate. Three ventilated male patients with pulmonary infiltrates on the intensive care unit were recruited after initial identification by the supervising medical and nursing team. The attending intensive care physician made an assessment of the capacity of each potential participant to make decisions about potential enrolment in the study. Those with capacity gave written acknowledgement of informed consent after receiving oral and written explanation of the study. For those without capacity, a personal legal representative (also known as guardian/welfare attorney/nearest relative) gave written acknowledgement of informed consent after receiving oral and written explanation of the study. Participants’ general practitioners were informed of their participation in the study. If a participant enrolled by the consent of their personal legal representative subsequently regained capacity they received a written explanation of the study. Six patients known to have bronchiectasis were recruited after initial identification by the supervising medical and nursing team. Participants gave written acknowledgement of informed consent after receiving oral and written explanation of the study. A full description of inclusion and exclusion criteria can be found in Supplementary table S6.

### Testing in humans

The safety of ascending doses of NAP (5 µg × 2, 10 µg × 2 and 80 µg × 2) was assessed in healthy volunteers. Each participant received only one dose of NAP, with the maximum dose within the microdosing limits set out by the European Medicines Agency (ICH Topic M3 (R2)). NAP was always delivered directly to the deep lung. Healthy volunteers were offered non-sedated bronchoscopy or received intravenous midazolam and alfentanil. Topical local anaesthesia (Lidocaine) was applied to the nasopharynx and flexible fibre-optic bronchoscopy proceeded per nasum or per orum. The bronchoscope was wedged in the right middle lobe (and additionally the right lower lobe for participants receiving 80 µg of NAP). NAP was administered via flexible catheter (1.5 mm APC catheter, ERBE, GA, USA) to the distal part of the lung in each lobe visited. OEM recording took place before and after the delivery of NAP. An independent data monitoring and safety committee reviewed all available clinical and laboratory information before providing permission to proceed with the next participant and also gave explicit permission to proceed to the Intensive Care Unit. Healthy volunteers remained in the Edinburgh Clinical Research Facility for 24 h after the administration of NAP for the purposes of clinical, radiological and laboratory monitoring and received a telephone follow-up phone call 72 h after the administration of NAP to enquire about the development of any symptoms that could have constituted an adverse event. Ventilated patients on the intensive care unit underwent chest x-ray to determine the most appropriate lobes to assess. For the procedure, patients received adequate sedation and analgesia, typically via the intravenous infusion of propofol and alfentanil. A physician provided peri-procedural anaesthesia for the patient. A bronchoscope was passed through the endotracheal tube to the pre-determined lobes. Otherwise, the administration of NAP and the alveoscopy imaging procedure were identical to the healthy volunteer study. Clinical, radiological and laboratory monitoring took place on the intensive care unit and each patient was reviewed thoroughly at 24 and 72 h after dosing to determine if any adverse events had occurred. Patients with bronchiectasis underwent sedated bronchoscopy. Lobes for investigation were selected by interrogation of available radiological imaging. If possible, a non-diseased lung segment was selected as a control. The delivery of NAP and imaging proceeded as per the healthy volunteer portion of the study, except where patients indicated discomfort or intolerance of the procedure, in which case the procedure was abbreviated at the discretion of the endoscopist. The initial imaging dose was always 24 µg per lobe, and the total delivered dose was always 80 µg. If participants were recruited from the bronchiectasis out patient clinic, they were followed up for 4 h in the dedicated medical day case unit. Discharge occurred on the basis of satisfactory observations and post-procedure chest x-ray appearances, and if standard medical day case unit discharge criteria were met. Participants were followed up by telephone at 24 h and 72 h. If participants were current in-patients on the respiratory ward, they returned there after bronchoscopy. They received the same follow-up but responsibility for other treatments and decisions returned to the attending clinical team.

### Human safety monitoring

For the purposes of screening, safety surveillance the following venous blood and urine samples were acquired. Haematology: haemoglobin, white cell count and platelet count. Biochemistry: sodium, potassium, urea, creatinine, bilirubin, ALT, glucose and C-reactive protein. Blood samples were analysed by the clinical laboratories of the Royal Infirmary of Edinburgh. Urinary drug screen: cocaine, amphetamines, morphine, benzodiazepines, methadone and THC (healthy volunteers only). The following additional safety measurements were performed. Vital signs: Blood pressure (Omron 705 BP machine), heart rate, pulse oximetry (Mindray, Shenzhen, China) temperature (Genius 2 IR Tympanic Thermometer, Covidien, Dublin, Republic of Ireland) and respiratory rate. Electrocardiogram: 12 lead (Mac 1200, GE Healthcare, Buckinghamshire, UK). Spirometry: FEV_1_, FVC and FEV_1_:FVC ratio (Alpha, Vitalograph, Buckingham, UK, healthy volunteers only). Chest X-ray: trial physician interpretation. Arterial blood gas analysis: PaO_2_, PaCO_2_, [H^+^], [HCO^3−^] and base excess (intensive care patients only, Rapidlab 1265, Siemens, Munich, Germany). Ventilatory parameters: Mode of ventilation, positive end expiratory pressure, ventilatory frequency (total and mandatory), tidal volume, and minute ventilation (intensive care patients only, Infinity C500 or Evita XL, Dräger). The schedule of study specific investigations can be found in the Supplementary material (Supplementary Tables S7, S8, and S9).

### Statistical analysis

Statistical analyses are described with each result. All statistical analyses were performed in Prism v6 (GraphPad Software, CA, USA), except Kappa statistics, which were calculated using the GraphPad online calculator, located at https://graphpad.com/quickcalcs/kappa1.cfm. An independent statistician confirmed that no statistical analyses were required to achieve the objectives of the exploratory clinical study.

### Supplementary Information

Supplementary Video 1.

Supplementary Video 2.

Supplementary Video 3.

Supplementary Information.

## Data Availability

Data are available from the corresponding author upon reasonable request.

## References

[1] Dorward DA (2017). The cyclin-dependent kinase inhibitor AT7519 accelerates neutrophil apoptosis in sepsis-related acute respiratory distress syndrome. Thorax.

[2] Donnelly SC (1995). Plasma elastase levels and the development of the adult respiratory distress syndrome. Am. J. Respir. Crit. Care Med..

[3] Wilkinson TS (2012). Ventilator-associated pneumonia is characterized by excessive release of neutrophil proteases in the lung. Chest.

[4] Donnelly SC (1993). Interleukin-8 and development of adult respiratory distress syndrome in at-risk patient groups. Lancet.

[5] Conway Morris, A. *et**al.* C5a mediates peripheral blood neutrophil dysfunction in critically ill patients. *Am.**J.**Respir.**Crit.**Care**Med.* **180,** 19–28 (2009). 10.1164/rccm.200812-1928OC PMC2948533 19324972

[6] Kolaczkowska E, Kubes P (2013). Neutrophil recruitment and function in health and inflammation. Nat. Rev. Immunol..

[7] de Oliveira, S., Rosowski, E. E. & Huttenlocher, A. Neutrophil migration in infection and wound repair: going forward in reverse. *Nat.**Rev.**Immunol.* **16** (2016). 10.1038/nri.2016.49 PMC5367630 27231052

[8] Segel GB, Halterman MW, Lichtman MA (2011). The paradox of the neutrophil’s role in tissue injury. J. Leukoc. Biol..

[9] Lee WL, Downey GP (2001). Neutrophil activation and acute lung injury. Curr. Opin. Crit. Care.

[10] Jia SH (2014). Activated neutrophils induce epithelial cell apoptosis through oxidant-dependent tyrosine dephosphorylation of caspase-8. Am. J. Pathol..

[11] Grommes J, Soehnlein O (2011). Contribution of neutrophils to acute lung injury. Mol. Med..

[12] Abraham E (2003). Neutrophils and acute lung injury. Crit. Care Med..

[13] Hoenderdos K, Condliffe A (2013). The neutrophil in chronic obstructive pulmonary disease. Am. J. Respir. Cell Mol. Biol..

[14] Chalmers JD (2012). Short- and long-term antibiotic treatment reduces airway and systemic inflammation in non-cystic fibrosis bronchiectasis. Am. J. Respir. Crit. Care Med..

[15] Gehrig S, Mall MA, Schultz C (2012). Spatially resolved monitoring of neutrophil elastase activity with ratiometric fluorescent reporters. Angew. Chem. Int. Ed. Engl..

[16] Charlton J, Sennello J, Smith D (1997). In vivo imaging of inflammation using an aptamer inhibitor of human neutrophil elastase. Chem. Biol..

[17] Craven TH (2018). Super-silent FRET sensor enables live cell imaging and flow cytometric stratification of intracellular serine protease activity in neutrophils. Sci. Rep..

[18] Kossodo, S. *et**al.* Noninvasive in vivo quantification of neutrophil elastase activity in acute experimental mouse lung injury. *Int.**J.**Mol.**Imaging* 581406, 10.1155/2011/581406 (2011). 10.1155/2011/581406 PMC3175392 21941648

[19] Akram, A. R. *et**al.* In situ identification of Gram-negative bacteria in human lungs using a topical fluorescent peptide targeting lipid A. *Sci.**Transl.**Med.* **10,** eaal0033 (2018). 10.1126/scitranslmed.aal0033 30355797

[20] Thiberville, L. *et**al.* Confocal fluorescence endomicroscopy of the human airways. 444–449 (2012). 10.1513/pats.200902-009AW 19687217

[21] Shafiek H (2016). Usefulness of bronchoscopic probe-based confocal laser endomicroscopy in the diagnosis of *Pneumocystis**jirovecii* pneumonia. Respiration.

[22] Thiberville L (2007). In vivo imaging of the bronchial wall microstructure using fibered confocal fluorescence microscopy. Am. J. Respir. Crit. Care Med..

[23] Avlonitis N (2013). Highly specific, multi-branched fluorescent reporters for analysis of human neutrophil elastase. Org. Biomol. Chem..

[24] Kawabata K (1991). ONO-5046, a novel inhibitor of human neutrophil elastase. Biochem. Biophys. Res. Commun..

[25] Moraes TJ, Zurawska JH, Downey GP (2006). Neutrophil granule contents in the pathogenesis of lung injury. Curr. Opin. Hematol..

[26] Fortunati E, Kazemier KM, Grutters JC, Koenderman L, den Bosch V, van JMM (2009). Human neutrophils switch to an activated phenotype after homing to the lung irrespective of inflammatory disease. Clin. Exp. Immunol..

[27] Jones H (1997). Dissociation of neutrophil emigration and metabolic activity in lobar pneumonia and bronchiectasis. Eur. Respir. J..

[28] Jones HA (1994). In vivo measurement of neutrophil activity in experimental lung inflammation. Am. J. Respir. Crit. Care Med..

[29] Lukawska JJ (2014). Imaging inflammation in asthma: real time, differential tracking of human neutrophil and eosinophil migration in allergen challenged, atopic asthmatics in vivo. EBioMedicine.

[30] de Prost N, Tucci MR, Melo MFV (2010). Assessment of lung inflammation with 18F-FDG PET during acute lung injury. AJR. Am. J. Roentgenol..

[31] Jevprasesphant R, Penny J, Attwood D, McKeown NB, D’Emanuele A (2003). Engineering of dendrimer surfaces to enhance transepithelial transport and reduce cytotoxicity. Pharm. Res..

[32] Doherty GJ, McMahon HT (2009). Mechanisms of endocytosis. Annu. Rev. Biochem..

[33] Segal AW, Geisow M, Garcia R, Harper A, Miller R (1981). The respiratory burst of phagocytic cells is associated with a rise in vacuolar pH. Nature.

[34] Cross AR, Segal AW (2004). The NADPH oxidase of professional phagocytes–prototype of the NOX electron transport chain systems. Biochim. Biophys. Acta.

[35] Jankowski A, Scott CC, Grinstein S (2002). Determinants of the phagosomal pH in neutrophils. J. Biol. Chem..

[36] Witko-Sarsat V, Rieu P, Descamps-Latscha B, Lesavre P, Halbwachs-Mecarelli L (2000). Neutrophils: Molecules, functions and pathophysiological aspects. Lab. Investig..

[37] Lee WL, Downey GP (2001). Leukocyte elastase: Physiological functions and role in acute lung injury. Am. J. Respir. Crit. Care Med..

[38] Levine AP, Duchen MR, de Villiers S, Rich PR, Segal AW (2015). Alkalinity of neutrophil phagocytic vacuoles is modulated by HVCN1 and has consequences for myeloperoxidase activity. PLoS ONE.

[39] Mitra S, Modi KD, Foster TH (2013). Enzyme-activatable imaging probe reveals enhanced neutrophil elastase activity in tumors following photodynamic therapy. J. Biomed. Opt..

[40] Arranz A, Ripoll J (2015). Advances in optical imaging for pharmacological studies. Front. Pharmacol..

[41] Kaiser E, Colescott RL, Bossinger CD, Cook PI (1970). Color test for detection of free terminal amino groups in the solid-phase synthesis of peptides. Anal. Biochem..

[42] Fischer R, Mader O, Jung G, Brock R (2003). Extending the applicability of carboxyfluorescein in solid-phase synthesis. Bioconjug. Chem..

[43] Haslett C (1985). Modulation of multiple neutrophil functions by preparative methods or trace concentrations of bacterial lipopolysaccharide. Am. J. Pathol..

