# Supplementary material for: Activated neutrophil fluorescent imaging technique for human lungs
Source: Sci Rep. 2021 Jan 13;11:976. doi: 10.1038/s41598-020-80083-w (PMC7806726; doi:10.1038/s41598-020-80083-w)
Supplement: Supplementary file 4 — Supplementary Information. [file 41598_2020_80083_MOESM4_ESM.pdf]

Activated neutrophil fluorescent imaging technique for human lungs

Thomas H Craven[1,2], Tashfeen Walton[3], Ahsan R Akram[1], Emma Scholefield[1], Neil McDonald[1], Duncan C Humphries[1], Bethany Mills[1], Thane A Campbell[1], Annya Bruce[1], Joanne Mair[1], Adam Marshall[1], James W Dear[4], David E Newby[4], Adam T Hill[1], Timothy S Walsh[2], Chris Haslett[1], Kevin Dhaliwal[1]

1 – Pulmonary Optical Molecular Imaging Group, Centre For Inflammation Research, University of Edinburgh

2 – Edinburgh Critical Care Research Group, University of Edinburgh

3 – School of Chemistry, EaStCHEM, University of Edinburgh

4 – Centre for Cardiovascular Science, University of Edinburgh

Correspondence to: : Dr T H Craven, The Centre for Inflammation Research, Queen's Medical Research Institute, University of Edinburgh, 47 Little France Crescent, Edinburgh EH16 4TJ, UK; thomas.craven@ed.ac.uk

SUPPLEMENTAL MATERIAL

SUPPLEMENTAL VIDEOS

S1 Laboratory confocal microscopy illustrating a time course of dequenching in treated PMNs.

S2 Optical endomicroscopy of alveolar tissue in a patient with bronchiectasis after receiving NAP (control segment).

S3 Optical endomicroscopy of alveolar tissue in a patient with bronchiectasis after receiving NAP (abnormal segment).

SUPPLEMENTAL RESULTS

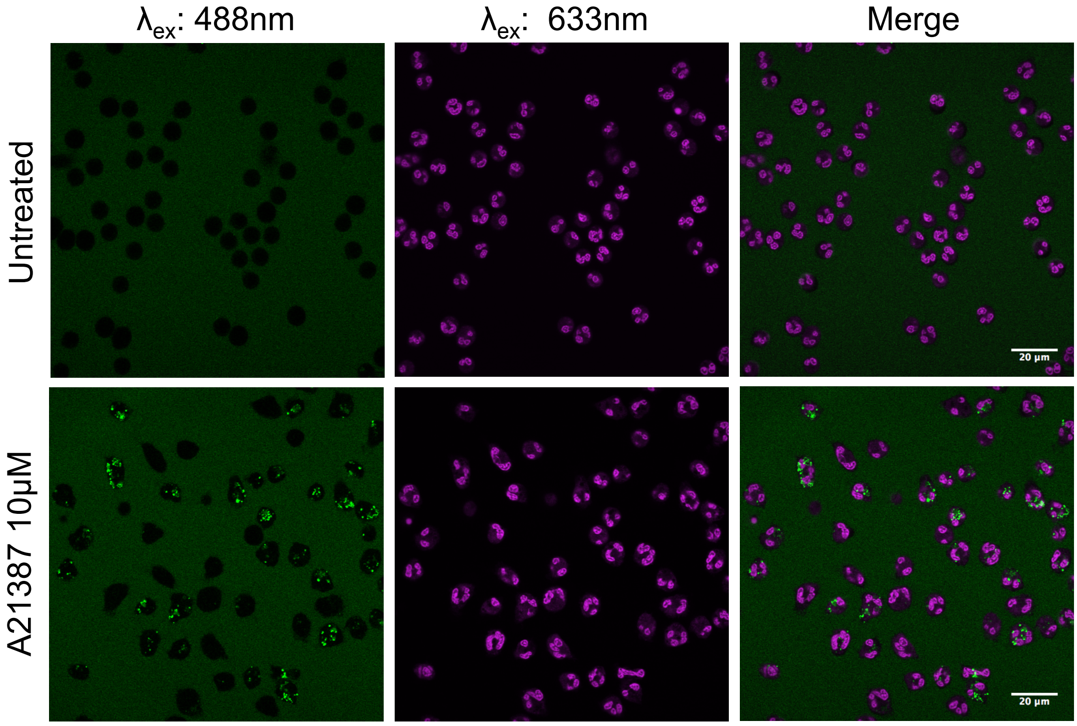

Supplemental figure S1. Untreated PMNs do not take up NAP. PMNs activated by A23187 Calcium Ionophore (10 μM) show intracellular green fluorescence. NAP is excited at 488 nm. Cells incubated with a nucleic acid counterstain (Syto60), which is excited at 633 nm. Images are representative of multiple fields of view across three independent experiments. Scale bar 20 μm.

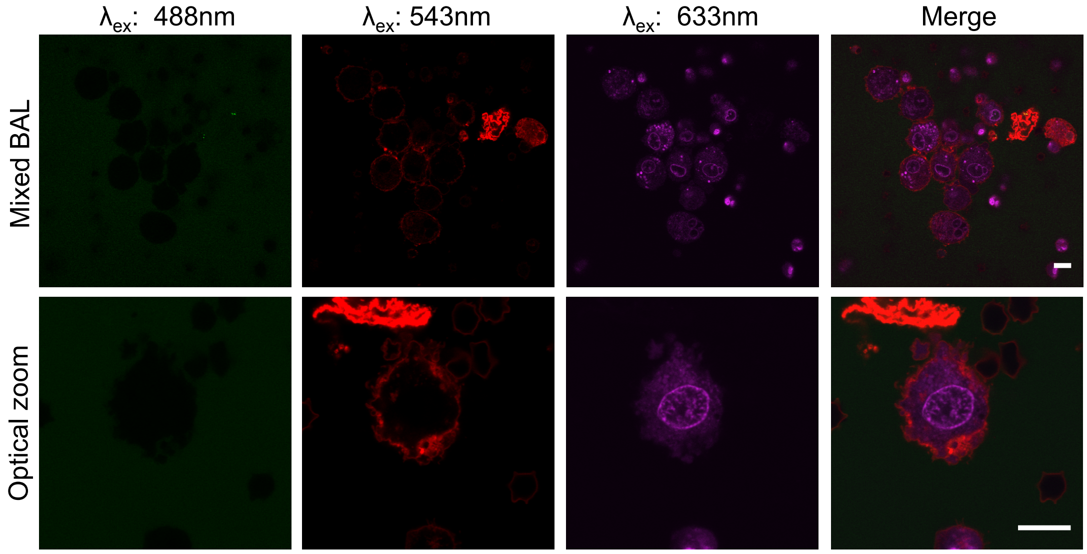

Supplemental figure S2. Confocal microscopy images of alveolar cells including alveolar macrophages from lavage from a patient with idiopathic pulmonary fibrosis. Cells have been exposed to NAP. There is no intracellular fluorescence. NAP is excited at 488 nm. A membrane stain is excited at 543 nm. Syto60, a nucleic acid stain, is excited at 633 nm. Top panel - wide field of view, bottom panel – optical zoom from different field of view. Scale bar 10 μM. Images are representative of three separate experiments.

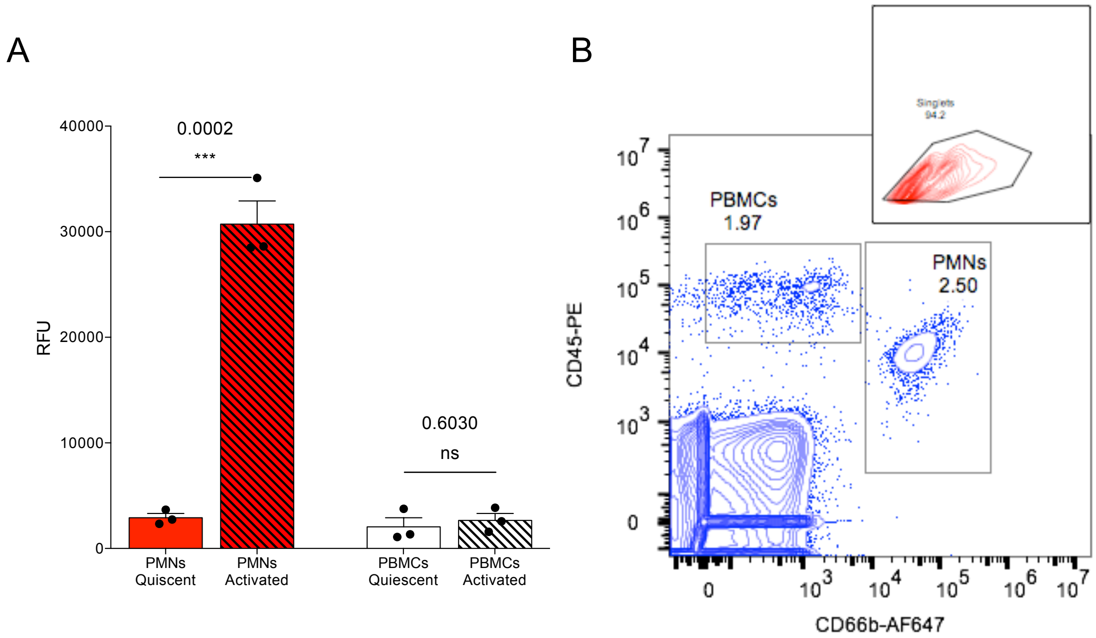

Supplemental figure S3. Flow cytometry of mixed leucocyte population. A) PMNs, activated by 1 µM A23187 (Calcium Ionophore) exhibit increased fluorescence in the FL1 channel (PMNs quiescent vs activated (mean MFI ±SEM): 2584 (217) vs 26993 (2021); PBMCs quiescent vs activated: 1771 (333) vs 2517 (245)). PBMCs (CD45++/CD66b-) and PMNs (CD45+/CD66b++) are distinguished from non-leucocyte components of erythrocyte free whole blood after the exclusion of doublets and other aggregates. Graph shows mean point estimate (±SEM) for n=4, Student's t-test, two tailed test, four degrees of freedom, exact p values shown, ***=p<0.001, ns=not significant. B) Example flow plot from one replicate, gate and percentage of parent gate shown (inset: parent gate).

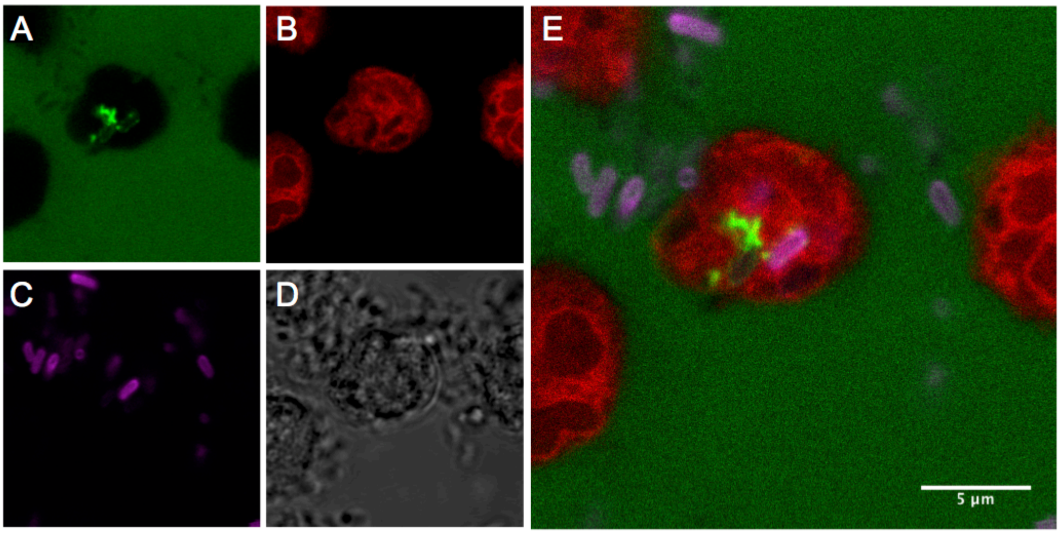

Supplemental figure S4. Co-localisation of green fluorescence and fluorescently labelled freshly cultured *Pseudomonas aeruginosa*. A) NAP fluorescence (λex 488 nm), B) Calcein Red cytoplasmic stain (λex 580 nm), C) Bacteria (Cell Vue Claret farred linker), λex 660 nm D) brightfield image E) Merge, scale bar as indicated. Images have been adjusted for presentation and are representative of repeated fields of view from repeated experiments.

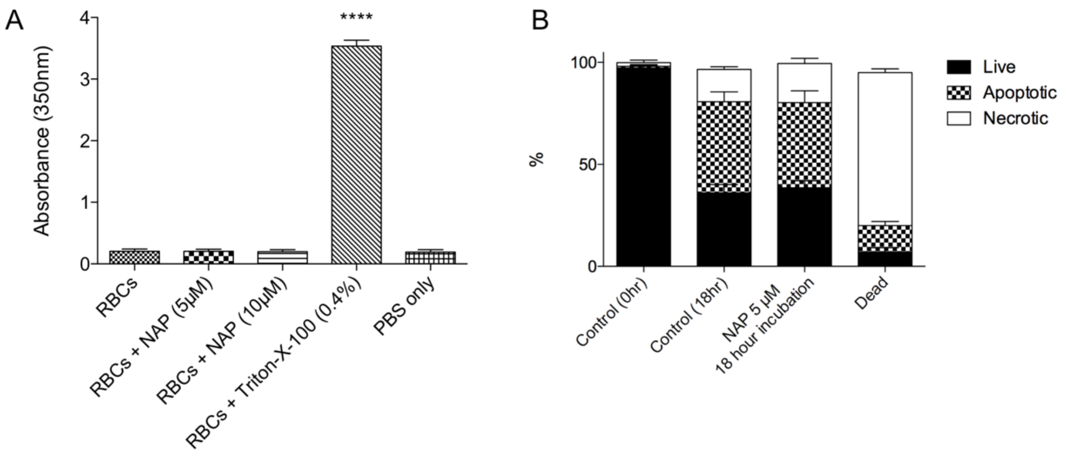

Supplemental figure S5 Cellular toxicity assessments with NAP. A) Membrane toxicity assessed using erythrocyte haemolysis. Triton is included as a positive control. Proportion of haemolysis is expressed as a percentage of the positive control. ****: $p < 0.0001$. B) Neutrophil apoptosis and necrosis with NAP. Expressed as percentage of all cells. No significant differences between control and NAP identified. N=3 both experiments.

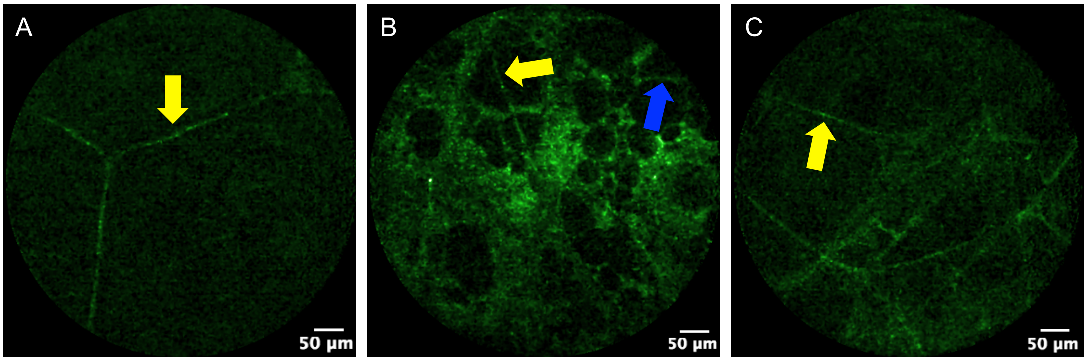

Supplemental figure S6. Still OEM images from healthy volunteers receiving NAP. A) Prior to the application of NAP. Lung autofluorescence permits the visualisation of lung structure. Alveolar walls are identified by the yellow arrows. B) Immediately after the application of NAP. Fluorescent bubbles can be seen (blue arrows), confirming the OEM tip has been re-positioned in a lung sub segment that has been exposed to NAP. C) Following the dissipation of NAP, the quantity of background fluorescence has reduced to allow for interrogation of the image for underlying punctate cellular fluorescent signal. None is seen in these healthy volunteers.

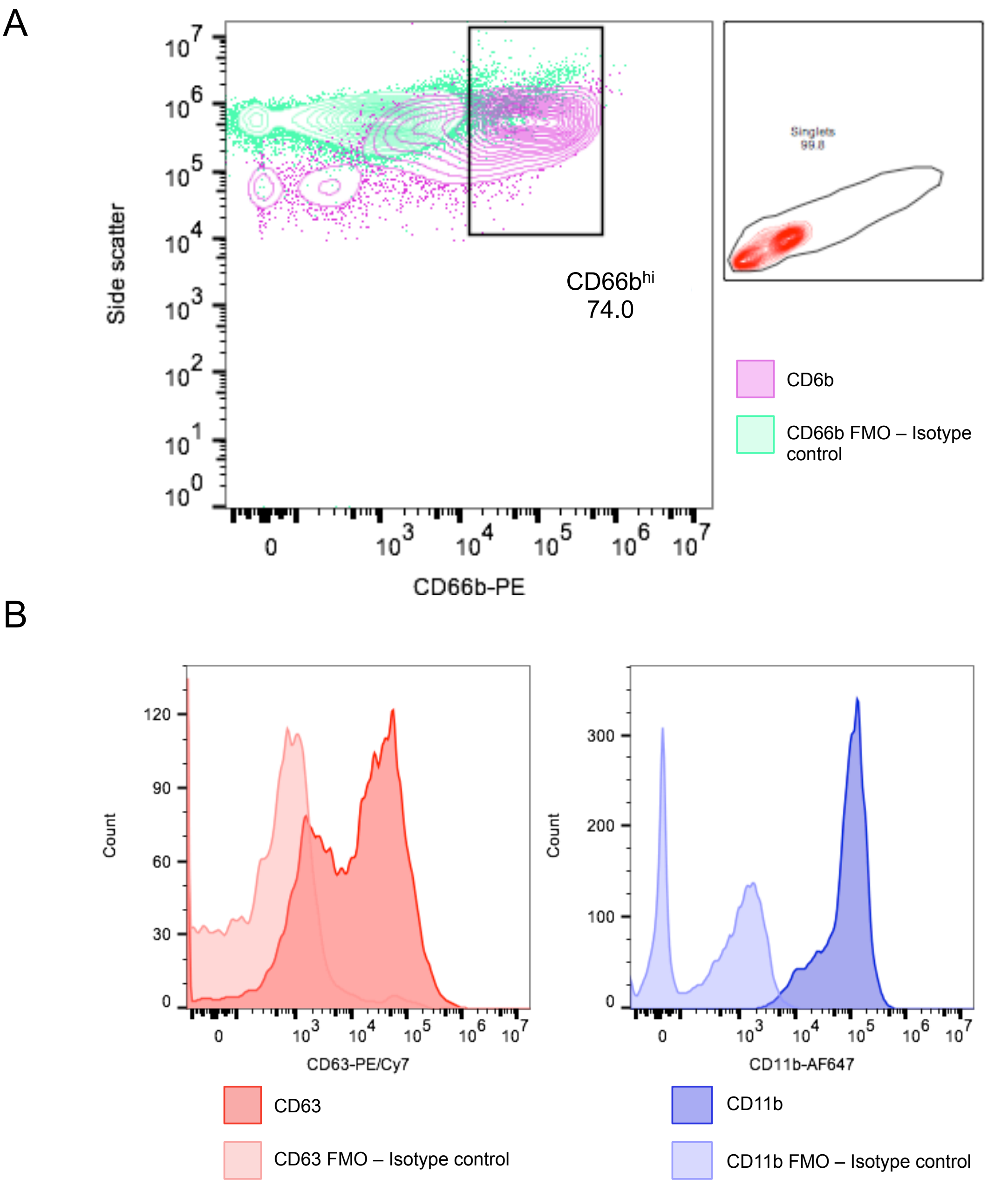

Supplemental figure S7. CD66^{hi} cells identified from broncho-alveolar lavage A) Flow cytometry gating strategy. Population of interest is shown with example percentage (inset: parent gate). The FMO-I control is overlaid and guides the position of the population gate. B) CD66^{hi} cells are forwarded for analysis. Example histograms indicating the ability to detect each surface marker distinct from its FMO-I (Fluorescence minus one – isotype) control.

**Timepoint**	**Appearance**	**NAP content (% area)**	**Related Substances (% area)**
Specification	Orange powder	Not less than 98%	Total ≤ 2%<br>Each impurity ≤0.5%
Initial (Aug 2011)	Complies	100	0
**2 month** (Oct 2011)<br>-20°C<br>2-8°C<br>25°C±3/75%±5 RH	 Complies<br>Complies<br>Complies	 100<br>100<br>100	 0<br>0<br>0
**5 month** (Jan 2012)<br>-20°C<br>2-8°C<br>25°C±3/75%±5 RH	 Complies<br>Complies<br>Complies	 100<br>100<br>100	 0<br>0<br>0
**24 months** (Aug 2013)<br>-20°C	 Complies	 100	 0
**4 years and 5 months** (January 2016)<br>-20°C	Complies	102.7	0

Supplemental table 1. Drug substance stability results.

**Timepoint (months)**	**Condition**	**Assay µg/mL**	**Related substances**	**pH**	**Appearance**	**Particles**
**0**	25°C/60% R.H.	64.8	N/D	7.42	Complies	Complies
	40°C/75% R.H.	64.8	N/D	7.42	Complies	Complies
**3**	25°C/60% R.H.	70.1	N/D	7.42	Complies	Complies
	40°C/75% R.H.	67.9	N/D	7.41	Complies	Complies
**6**	25°C/60% R.H.	66.0	N/D	7.37	Complies	Complies
	40°C/75% R.H.	64.7	N/D	7.39	Complies	Complies

Supplemental table S2. Stability results for aqueous drug product. R.H. Relative Humidity. 40˚C/75% R.H. indicates accelerated stability confirming 2-year stability at room temperature and humidity.

Attribute		Method	Acceptance criteria
Description		Visual observation	Orange powder
Identification	(1)	IR Absorption	Conforms to the reference spectrum
	(2)	UV Absorption	Conforms to the reference spectrum
	(3)	MALDI-ToF spectroscopy	Is concordant with that of the reference material
Melting point		Melting point apparatus	Decomposition after 230°C
Purity	(1) NAP content/Assay	HPLC	Greater than 98%
	(2) Related substances	HPLC (UV and ELSD)	Each: ≤0.5%
	(3) Heavy metals	ICP-MS	Total: 30.0 ppm Class 1 elements
	(4) Residual solvent (acetonitrile)	Headspace GC-MS	Acetonitrile: 410 ppm (Option 1)

Supplemental table 3. Release specifications for drug substance.

Test (method)	Acceptance criteria
Description (visual observation)	Light orange-tinged transparent solution with no visible particulates
Particles	Free from visible particles
Concentration	80 µg +/- 25% in 5mL
Identification of NAP (by HPLC)	The retention time of the principal peak in the sample chromatogram corresponds to that of the principal peak in the standard chromatogram
NAP content (by HPLC)	≥75% and ≤125% label claim
pH	7.30-7.50

Supplemental table S4. Release specifications of aqueous drug product.

Subject number	Dose of NAP (µg)	Description of event	SAE	Causality	Severity
2	5	Felt faint after venepuncture	No	Unrelated	Mild
3	5	Fever	No	Unrelated	Mild
4	10	Fever	No	Unrelated	Mild
5	80	Sore throat	No	Unrelated	Mild
6	80	Sprained wrist after screening before dosing visit	No	Unrelated	Mild
9	80	Required surgery for pre-existing abdominal wall haematoma	Yes	Unrelated	Mild
9	80	Fever	No	Unrelated	Mild
12	80	Shortness of breath	No	Unrelated	Mild
15	80	Chest wall discomfort	No	Unrelated	Mild

Supplemental table S5. A list of recorded adverse events. All adverse events were either expected after standard bronchoscopy or clearly unrelated to the study procedure. No AE was deemed to be related to NAP.

<table>
<tr><td colspan="2">Inclusion Criteria</td></tr>
<tr><td>Healthy volunteers</td><td>Healthy male volunteers aged between 18 and 40</td></tr>
<tr><td>Intensive Care patients</td><td>Ventilated male patients over the age of 18 in the ICU</td></tr>
<tr><td>Patients with bronchiectasis</td><td>Male patients diagnosed with bronchiectasis over the age of 18</td></tr>
<tr><td colspan="2">Exclusion Criteria</td></tr>
<tr><td>Healthy volunteers</td><td>1. Age < 18 or >40 years

2. History of any chronic or ongoing acute illness (with particular reference to asthma, upper respiratory tract infection, lower respiratory tract infection, bronchiectasis, congenital heart disease, ischaemic heart disease, valvular heart disease, diabetes mellitus, chronic renal impairment, urinary tract infection)

3. Any current medication

4. Any history of previous reactions to fluorescein or any other anaphylaxis

5. Abnormal physical signs detected at cardiorespiratory examination

6. Temperature >37.3 degrees Celsius

7. Oxygen saturation <95% breathing room air

8. Haemoglobin, white cell count or platelet count outside the normal laboratory reference range

9. Blood sodium, potassium, urea, creatinine, bilirubin, alanine aminotransferase, random glucose or C-reactive protein outside the normal laboratory reference range

10. Forced expiratory volume in one second (FEV1) or forced vital capacity (FVC) <80% predicted

11. FEV1:FVC ratio <70%

12. Any significant cardiorespiratory abnormality detected on chest x-ray

13. Peripheral venous access insufficient to support 14 gauge cannulae.

14. General practitioner confirmation of eligibility as a healthy volunteer not received

15. Failure to provide suitable identification (passport/driving license)

16. Refusal to consent to enter details in ‘The Over Volunteering Prevention System’ (TOPS) database

17. Positive urine drug screen

18. Participation in any other interventional study or less than three months since their last participation in an interventional study

19. Female</td></tr>
</table>

Intensive Care patients	1. Age <18 years<br><br>2. Any contraindication for bronchoscopy<br><br>3. Refusal for participation by attending consultant<br><br>4. Fi02 >70%<br><br>5. PEEP>10 cm<br><br>6. Recent pneumothorax (whilst on ventilator)<br><br>7. Any history of previous reactions to fluorescein or any other anaphylaxis<br><br>8. Participation in any other interventional study or less than three months since their last participation in an interventional study<br><br>9. Female
Patients with bronchiectasis	1. Age <18years<br><br>2. Any contraindication for bronchoscopy<br><br>3. Refusal for participation by attending consultant<br><br>4. Recent pneumothorax (last 4 weeks)<br><br>5. Myocardial infarction within the preceding 4 weeks<br><br>6. Any history of previous reactions to fluorescein or any other anaphylaxis<br><br>7. Participation in any other interventional study or less than three months since their last participation in an interventional study<br><br>8. Female

Supplemental table S6. Inclusion and exclusion criteria for enrolment in the Phase 1 study.

Healthy volunteers							
Investigation	Day of screening	Study day on arrival	Following NAP delivery	1 hour following NAP delivery	4 hours following NAP delivery	Overnight	24 hours (± 30 mins)
Informed Consent	⊗						
Cardio-respiratory examination	⊗	⊗			⊗		⊗
Vital signs⦿	⊗	⊗	⊗	⊗	⊗	⊗	⊗
Venous blood samples	⊗	⊗			⊗		⊗
Spirometry	⊗	⊗			⊗		⊗
Chest x-ray	⊗						⊗
Venous blood for IMP detection (non safety)				⊗			
Urine collection for IMP detection (non-safety)°			⊗				

⦿ Vital sign monitoring was performed hourly for the duration of the healthy volunteers attendance at the Edinburgh Clinical Research Unit. It was permissible to suspend vital sign monitoring overnight if the volunteer was sleeping comfortably.

° The first and second voided urine volumes following the administration of NAP were collected for analysis.

Supplemental table S7. Schedule of investigations for healthy participants

Participants with bronchiectasis						
Investigation	Day of screening	Study day on arrival	1 hour (+/- 30 mins)	4 hours (+/- 30 mins)	24 hours (+/- 4 hours)	72 hours (± 4 hours)
Informed consent	⊗					
Permission from attending physician	⊗					
Chest X-ray	⊗			⊗		
Bloods: FBC, U&E's, LFTs, CRP	⊗			⊗		
Cardiorespiratory examination	⊗	⊗		⊗		
Observations, pulse, temperature, oxygen saturation	⊗	⊗	⊗	⊗		
Venous Blood For IMP (non-safety)			⊗			
Urine Collection for IMP (non-safety)			⊗	⊗		
Telephone follow-up					⊗	⊗

Supplemental table S8. Schedule of investigations for participants with bronchiectasis

ITU participants						
Investigation	Day of screening	Immediately pre-procedure	7.5 minutes following NAP delivery	2 hour following NAP delivery	6 hours following NAP delivery	24 hours (± 30 mins)
Informed Consent	⊗					
Permission from attending physician	⊗					
Cardio-respiratory examination	⊗					
Vital signsϕ	⊗	⊗		⊗	⊗	⊗
Venous blood samples	⊗			⊗	⊗	⊗
Arterial Blood Gas		⊗		⊗	⊗	⊗
Ventilator settings	⊗	⊗		⊗	⊗	⊗
Chest X-ray	⊗	⊗				⊗
Venous blood for IMP detection (non- safety)			⊗			
Urine collection for IMP detection (non-safety)			⊗	⊗		

ϕ Patients were continuously monitored in keeping with standard practice

Supplemental table S9. Schedule of investigations for participants from ITU
